# Modulating the Immunosuppressive Tumor Microenvironment and Inhibiting Growth in Mutp53-Driven CRPC via STAT3 Pathway Blockade

**DOI:** 10.7150/ijbs.111732

**Published:** 2025-04-22

**Authors:** Zichen Bian, Jia Chen, Yufan Wang, Chen Jin, Chaozhao Liang, Jialin Meng, Meng Zhang

**Affiliations:** 1 Department of Urology, The First Affiliated Hospital of Anhui Medical University; Institute of Urology, Anhui Medical University; Anhui Province Key Laboratory of Urological and Andrological Diseases Research and Medical Transformation, Anhui Medical University, Hefei, 230022, China.

**Keywords:** castration-resistant prostate cancer, *TP53*, p.R248Q, immune-suppressed microenvironment, STAT3 signaling

## Abstract

Mutations in *TP53*, particularly the p.R248Q variant, contribute to the progression of castration-resistant prostate cancer (CRPC) by reshaping the tumor microenvironment (TME). This study examined the impact of p.R248Q (mutp53) on immune suppression and CRPC progression. We introduced the *Trp53* p.R245Q mutation into RM-1 mouse prostate cancer (PCa) cells via CRISPR/Cas9, which mimics human *TP53* p.R248Q. These cells were implanted into C57BL/6 mice to model tumor progression and immune interactions. Mice were treated with JAK2 and STAT3 inhibitors to assess immune and tumor responses. Tumor behavior and immune responses were analyzed via histology, immunofluorescence, flow cytometry, Enzyme-linked immunosorbent assay (ELISA), and bioinformatics. Findings were validated in the C4-2 human PCa cell line. Compared with wild-type p53, *TP53* mutations were present in 27% of PCa patients and were significantly correlated with reduced overall survival (p < 0.001, HR = 1.97) and recurrence-free survival (p = 0.02, HR = 1.62). The p.R248Q mutation was most prevalent. Gene-edited mutp53 cells exhibited increased proliferation and tumorigenicity. Screening and validation confirmed that IL6/JAK2/STAT3 pathway activation in mutp53 tumors led to immune microenvironment alterations. Flow cytometry and immunofluorescence revealed an immunosuppressive profile, with decreased proinflammatory cytokines and elevated anti-inflammatory factors. Coimmunoprecipitation revealed that mutp53 competes with SHP1 for STAT3 binding, sustaining its activation. Inhibition of STAT3 reduced mutp53-driven immune suppression and tumor progression. Mutp53 promotes an immunosuppressive TME and facilitates CRPC progression through the STAT3 pathway, underscoring its potential as a therapeutic target.

## 1. Introduction

Prostate cancer (PCa) poses a major health challenge for middle-aged and elderly men, ranking as the third most common malignancy in males. Its burden has been steadily increasing, correlating with economic development 1-3. PCa exhibits significant heterogeneity, with cases varying from slow-growing to highly aggressive forms 4. The emergence of castration-resistant prostate cancer (CRPC) poses a significant therapeutic obstacle, with accumulating evidence highlighting the pivotal contribution of *TP53* gene alterations in this transformation 5-10. In PCa, *TP53* mutations frequently coincide with TMPRSS2-ERG fusions, jointly accelerating malignancy through the activation of β-catenin-mediated pyrimidine biosynthesis 8. Furthermore, EZH2 has been shown to associate with the internal ribosome entry site (IRES) of p53 mRNA, selectively enhancing the translation of gain-of-function (GOF) p53 variants, thereby facilitating tumor progression and metastatic potential 9. In addition, mutant p53 (Mutp53) suppresses DAB2IP, a known tumor suppressor, leading to amplified insulin-stimulated AKT1 signaling, which in turn augments cellular proliferation and invasiveness in PCa 10.

In cancer research, nearly 80% of *TP53* mutations are missense variants that impact the DNA-binding domain, frequently accumulating at distinct hotspot regions 11-14. These mutations produce a full-length p53 protein but disrupt its native tumor-suppressive function 15, 16. Additionally, many p53 missense mutant lose their tumor-suppressive function and simultaneously GOF properties 12, 17. Findings from both *in vitro* and *in vivo* mouse studies suggest that these missense mutations exhibit stronger oncogenic potential compared to p53-null variants 15, 17-19. Mutp53 disrupts normal transcriptional regulation by aberrantly interacting with other transcription factors, thereby driving tumor-promoting gene expression programs 8, 20-24. An alternative theory suggests that the persistence of *TP53* missense mutations might be explained by their dominant-negative effects, which compromise the function of any remaining wild-type allele 25-29. A significant focus has been placed on the role of Mutp53 in driving cell migration, invasion, and metastasis in epithelial cancers such as PCa 29, and the impact of *TP53* missense mutations on cancer progression via modulation of the tumor microenvironment (TME) has yet to be fully explored. TME plays a critical role in PCa progression and therapeutic resistance, with increasing evidence highlighting the impact of immune suppression, stromal interactions, and cytokine-mediated signaling in shaping tumor aggressiveness 30-32. In a comprehensive cohort analysis of 1,557 PCa patients, we identified three immune subtypes: immune-activated, immune-suppressed, and nonimmune 33. Notably, *TP53* p.R248Q mutations were predominantly found in immune-suppressed and nonimmune subgroups but were absent in the immune-activated subgroup, indicating a potential role in shaping the immune landscape. This observation led us to hypothesize that p.R248Q contributes to an immunosuppressive TME by disrupting immune surveillance mechanisms, thereby facilitating tumor progression. The mouse sourced *Trp53* mutated cell lines or models were widely applied in the experimental to represent the impact of *TP53* mutation in human beings 34, 35. Several published studies also reported that *Trp53*^p.R245Q^are homologous to human common *TP53*^p.R248Q^ mutation. In this study, we established *Trp53*^p.R245Q^ mouse cell lines, which correspond to the human hotspot mutant *TP53*^p.R248Q^ 36-38. This mutation intensified immune suppression by enhancing JAK2/STAT3 signaling, thereby facilitating tumor progression. These insights are crucial for refining and advancing immunotherapeutic approaches for PCa treatment. In our study, we focused on the *TP53* p.R248Q mutation, which has greater gain-of-function than the other mutants do, as it was found to be more prevalent in both the immunosuppressive and nonimmunosuppressive subgroups (**Table S1**). Targeting the JAK2/STAT3 pathway in patients with *TP53* p.R248Q mutations may increase the efficacy of immunotherapies by reversing the immunosuppressive environment, offering a promising therapeutic strategy for advanced CRPC.

## 2. Materials and methods

### 2.1 Cell culture and maintenance

Human HEK293T cell cultures were maintained in DMEM (Shanghai VivaCell Biosciences, Ltd., Shanghai, China; cat. no. C3103-0500) supplemented with 10% fetal bovine serum (FBS) (Shanghai VivaCell Biosciences, Ltd., Shanghai, China; cat. no. C04001-500) and 1% penicillin/streptomycin (Proteintech Group, Inc., Wuhan, China; cat. no. PR40022). The human PCa cell line C4-2 and the murine cell line RM-1 were cultured in RPMI-1640 medium (Shanghai VivaCell Biosciences, Ltd., Shanghai, China; cat. no. C3001-0500) supplemented with 10% fetal bovine serum and 1% penicillin‒streptomycin. HEK293T cells were grown in DMEM supplemented with the same supplements. All cultures were maintained at 37°C in a 5% CO2 humidified atmosphere. The cell lines were obtained from the Cell Bank of the Chinese Academy of Sciences, located in Shanghai, China, and were verified to be mycoplasma free via routine polymerase chain reaction (PCR) testing. The cells were maintained within a passage number range of 5 to ensure consistency across experiments. The culture media of the RM-1 cells were changed every day, and those of the C4-2 and 293T cells were changed every 48 h to maintain optimal growth conditions and cell viability.

### 2.2 CRISPR/Cas9 gene editing techniques

We employed CRISPR/Cas9-mediated point mutagenesis to introduce the *Trp53* p.R245Q mutation (analogous to human *TP53* p.R248Q) in PCa cells via the Ubigene Biosciences platform. The cells were electroporated with CRISPR-U™ and Donor plasmids, followed by neomycin selection. Genomic DNA was extracted, and PCR was performed to verify genetic modifications, with successful edits confirmed by sequencing. Clones were expanded and cryopreserved. Additionally, CRISPR/Cas9-mediated knockout of the *TP53* gene in C4-2 cells was performed via the YCas-LV002 vector, with three specific sgRNAs inserted into the YKO-LV003-h*TP53*-neomycin vector. After transduction, the cells were selected with G418, and p53 protein depletion was confirmed through western blotting. Supplementary Table 1 contains the primer details, whereas the sgRNA sequences can be found in the Supplementary Materials and Methods.

### 2.3 Molecular biology techniques

Plasmids, transfections, and viral infections: siRNAs were obtained from Sangon Biotech and introduced into the cells via the Lipofectamine 3000 reagent (Invitrogen Corporation, Carlsbad, CA, USA; cat. no. L3000001) according to the manufacturer's protocols (for primer sequences, see **Supplementary Table 2**). The assays were conducted in triplicate across independent experiments, each confirming the observed protein interactions and expression levels. The overexpression plasmid for flag-*STAT3* (NM_139276) was constructed via the Ubi-MCS-3FLAG-SV40-EGFP-IRES-Hygromycin vector, whereas the wild-type *TP53* [NM_000546 (HA tag)] and mutant *TP53* [NM_000546 (R248Q) (HA tag)] plasmids were built into a CMV enhancer-MCS-SV40-puromycin vector. Enzymatic digestion, followed by PCR amplification, gel electrophoresis, and Sanger sequencing, was conducted to confirm the successful construction of these plasmids, which were generated by GeneChem (GeneChem Co., Ltd., Shanghai, China). The successful integration of these plasmids was confirmed via western blotting.

Western Blotting and Co-Immunoprecipitation (Co-IP): Cell lysates from C4-2, RM-1, and HEK293T cells were prepared on ice using lysis buffer (Cell Signaling Technology, MA, USA; cat. no. 9803) supplemented with protease and phosphatase inhibitors (Beyotime Biotech, Beijing, China; cat. no. P1008 and P1081). Protein samples (10 µg per lane) were separated using SDS-PAGE (180 V, 40 min), then transferred onto PVDF membranes (Merck, NJ, USA; cat. no. IPVH00010) via a wet blotting system (Bio-Rad, CA, USA; 350 mA, 90 min). Membranes were blocked in 5% BSA (TBST containing 0.1% Tween-20) for 20 min at room temperature and incubated overnight at 4°C with primary antibodies (1:1000 dilution). After washing with TBST, membranes were incubated with HRP-conjugated secondary antibodies (1:5000 dilution), and protein bands were detected using chemiluminescence on a Chemiscope 5600 analyzer (Clinx Science Instruments, Shanghai, China).

Co-IP: Co-IP experiments were performed using a commercial kit (Thermo Fisher Scientific, San Diego, CA, USA; cat. no. 26147) according to the manufacturer's instructions. Briefly, total protein (1000 µg per sample) was precleared with control agarose beads at 4°C for 50 min. Protein A/G Plus agarose beads were incubated with antibodies (10 µg each) against STAT3, phosphorylated STAT3, SHP1, HA, or Flag at room temperature with gentle agitation for 30 min. IgG served as a negative control. Antibodies were crosslinked onto beads using 450 µM disuccinimidyl suberate under rotation at room temperature for 50 min to ensure stability. Subsequently, precleared protein lysates were incubated overnight at 4°C with antibody-conjugated beads to facilitate antigen immunoprecipitation. The following day, the antigens were eluted, and the associated proteins were characterized via western blotting. Additional details on the antibodies utilized are provided in **Supplementary Table 3**. The assays were performed in three independent experimental replicates, each confirming the observed protein interactions and expression levels.

### 2.4 Cell proliferation and colony formation assays

Cell proliferation was determined by performing an MTT assay (3-[4,5-dimethyl-2-thiazolyl]-2,5-diphenyl-2-H-tetrazolium bromide) according to the manufacturer's protocol (Beyotime Biotech, Beijing, China; cat. no. C0009S). Briefly, control and treated C4-2 and RM-1 cells were cultured in 24-well plates and incubated with MTT reagent (50 µL, 5 mg/ml) for 2 h. After incubation, cells were transferred into 96-well plates, and absorbance (optical density, OD) was measured at 570 nm using a TECAN spectrophotometer (Tecan Group Ltd., Männedorf, Switzerland). Each experiment was independently repeated three times in triplicate.

For the colony formation assay, control and treated RM-1 and C4-2 cells were seeded in 6-well plates and cultured for eight days at 37°C. Colonies were then fixed with 4% paraformaldehyde, stained using 0.1% crystal violet, and counted microscopically if colonies contained over 50 cells. All experiments were independently replicated three times.

### 2.5 Animal studies and histological techniques

Mouse and Tumor Model Studies: Six-week-old male C57BL/6 mice from GemPharmatech Co., Ltd. (Nanjing, China) were used in this study. All animals were maintained in specific pathogen-free conditions at accredited facilities, and the study adhered to ARRIVE guidelines 39. Mice were randomly allocated to experimental groups, ensuring age matching and the absence of statistically significant differences in body weight among the groups. For the second batch, mice with established Mutp53 tumors were further randomized into treatment and control groups, and the tumor size was considered before randomization to ensure comparability. All animal handling, including husbandry and weighing, was performed by technicians blinded to the group assignments, with the final data compiled for analysis by the researchers.

For the RM-1 PCa model, 1 × 10⁶ RM-1 cells suspended in 100 μL of a 1:1 mixture of Matrigel (Shanghai Nova Pharmaceutical Technology Co., Ltd., Shanghai, China; cat. no. 0827045) and phosphate-buffered saline (PBS) were injected subcutaneously into the right flank of C57BL/6 mice. Mice were chosen from both the WTp53 and mutp53 groups to ensure similar tumor sizes of approximately 100 mm^3^. Subsequent treatment procedures were subsequently initiated. The respective treatments included a STAT3 inhibitor (10 mg/kg, orally administered every other day for 7 days; MedChemExpress LLC., NJ, USA; cat. no. HY-100753) and a Jak-2 inhibitor (20 mg/kg, orally administered daily for 13 days; MedChemExpress LLC., NJ, USA; cat. no. HY-131906). The treatment was terminated on day 21. The mice were then sacrificed, and the tumors were excised, photographed, and weighed.

Histological analysis was conducted using hematoxylin and eosin (H&E) staining on paraffin-embedded tissues, followed by dewaxing and sequential rehydration in xylene and graded alcohol solutions. Immunohistochemistry (IHC) was performed with p-JAK2 (1:50, Affinity Biosciences Group Ltd., Changzhou, China, cat. no. AF3024) and p-STAT3 (1:50; Cell Signaling Technology, Cell Signaling Technology, Inc., MA, USA; cat. no. # 9145) antibodies. Additionally, immunofluorescence staining was performed with primary antibodies against CD86 (1:1000; Cell Signaling Technology, Inc., MA, USA; cat. No. 91882), CD163 (1:1000; Abcam plc, Cambridge, UK; cat. EPR19518), CD45 (1:1000; Affinity Bioscience Ltd., Jiangsu, China; cat. No. DF6839), and IFN-γ (1:500; Wuhan Servicebio Technology Co., Ltd., Wuhan, China; cat. No. GB11107-1).

Flow cytometry: Single cells were isolated from subcutaneous tumor tissues and stained sequentially with fluorescently labeled antibodies. The primary antibodies used included those against IFN-γ (BD Biosciences, San Jose, CA, USA; cat. no. 554412), F4/80 (BioLegend, San Diego, CA, USA; cat. no. 123116), and CD11b (BioLegend, San Diego, CA, USA; cat. no. 101206). Additionally, antibodies against CD86 (Elabscience, Wuhan, China; cat. no. 213111) and CD206 (BD Biosciences, San Jose, CA, USA; cat. no. 565250) were utilized. Data acquisition was conducted via a CytoFLEX LX flow cytometer (Beckman Coulter, Brea, CA, USA), and the data were analyzed with FlowJo software (version 10.0, FlowJo LLC, Ashland, OR, USA). The flow cytometry experiments were conducted three times independently, with each experiment including samples from separate biological replicates.

Enzyme-linked immunosorbent assay (ELISA): Cytokine levels in prostate tumor tissue homogenate samples were detected via ELISA kits specific for IFN-γ (Elabscience, Wuhan, China; cat. no. E-EL-M0048), IL-10 (Servicebio, Wuhan, China; cat. GEM0003-96T), TGF-β (Elabscience, Wuhan, China; cat. no. E-UNEL-M0099), and IL-1β (Elabscience, Wuhan, China; cat. no. E-EL-M0037). The cytokine measurements via ELISA were also independently replicated three times, with each replicate including technical duplicates to confirm consistency.

### 2.6 Clinical data acquisition and bioinformatics analysis

We conducted a comprehensive retrospective analysis via cBioPortal 40 to assess the prevalence of *TP53* mutations in PCa, analyzing data from 7,678 patients across 19 studies, with follow-up data for 671 patients. Expression profiles and prognostic data from the GSE25236 41 and GSE107299 42, 43 cohorts were examined to evaluate SHP1 gene expression correlations. Transcriptomic sequencing was performed on murine xenografts of RM-1-derived WTp53 and mutp53 cells, followed by RNA isolation, mRNA enrichment, cDNA synthesis, library construction, and sequencing on an Illumina HiSeqTM 2500 platform 44, 45. Differential gene expression and relevant enrichment analyses were conducted via the "limma" and "ClusterProfiler" packages in R 46-49, and immune cell infiltration was assessed via the seq-ImmuCC platform 50. The methods are described in the **Supplementary Methods.**

### 2.7 Statistical analysis

Cox regression models were employed to evaluate survival rates and recurrence-free survival across different patient cohorts. Kaplan-Meier analysis was conducted to visualize patient survival outcomes 51, while the Weibull hazard model assessed the impact of *TP53* mutations in PCa patients. Pathway activities among subgroups were illustrated using the "ComplexHeatmap" package 52. Pearson correlation coefficients and chi-square tests were utilized to analyze continuous and categorical variables, respectively. Student's t-test and Kruskal-Wallis analyses were applied for subgroup comparisons involving two or multiple groups. All statistical tests were two-tailed, and significance was set at p < 0.05.

## 3. Results

### 3.1 Prevalence and clinical implications of *TP53* mutations in PCa

**Figure 1:**
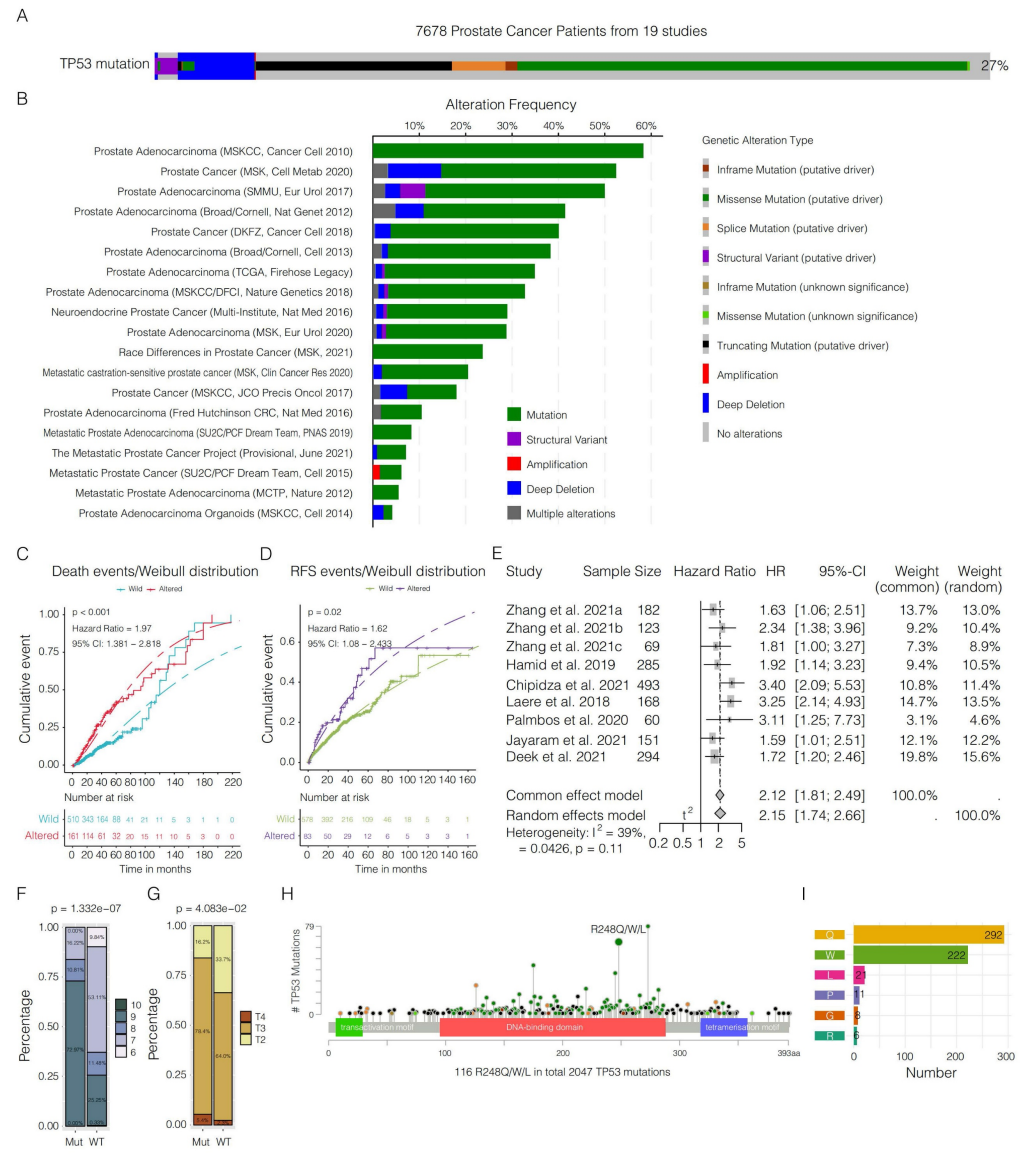
** Comprehensive analysis of *TP53* mutations in prostate cancer (PCa). A.** Overview of *TP53* mutation prevalence in 7,678 PCa patients from 19 different studies. **B.** Visualization of the frequency of various genetic alterations—such as mutations, structural variants, amplifications, deep deletions, and complex alterations—across multiple studies. **C.** Weibull distribution curve comparing the survival probability over time between patients with wild-type and altered *TP53* (p < 0.001, hazard ratio (HR) = 1.97, 95% CI = 1.381--2.818). **D.** Weibull distribution curve depicting recurrence rates over time for patients with and without *TP53* alterations (p = 0.02, hazard ratio (HR) = 1.62, 95% CI = 1.08--2.433).** E.** Meta-analysis of combined hazard ratios from various studies reinforcing the association between *TP53* mutations and the severity of PCa (HR = 2.15, 95% CI = 1.74-2.66, p < 0.01). **F‒G.** Analysis of Gleason scores (chi-square test, p = 1.332e‒07; **F**) and pathological T stages (chi-square test, p = 4.083e‒02; **G**) comparing distributions between the wild-type and mutant *TP53* patient groups. **H.** Examination of the distribution and prevalence of specific *TP53* mutations within the PCa patient cohort. **I.** Detailed breakdown of alterations in the R248 allele of *TP53*, providing insights into the impact of the mutation within the study cohort.

In our comprehensive synthesis of genetic data from 7,678 PCa patients across 19 studies, we documented a *TP53* mutation prevalence of approximately 27% (**Figure 1A-B, Supplementary Table 4**), with missense mutations being most frequent. This difference was especially pronounced in relation to patient survival and recurrence rates. Patients with *TP53* mutations exhibited markedly reduced survival rates (**Figure 1C**) and higher recurrence rates (**Figure 1D**)*.* A meta-analysis confirmed the association between *TP53* mutations and increased severity of advanced PCa (**Figure 1E, Supplementary Table 5**). These mutations were also correlated with more advanced PCa, as evidenced by higher Gleason scores (**Figure 1F**) and pathological T stages (**Figure 1G**). Among the *TP53* mutations, hotspot mutations at codons 273, 248, and 175 were especially prevalent (**Figure 1H**). Specifically, at the R248 site, the most frequent substitution was amino acid Q (**Figure 1I**).

On the basis of our recent study 33, we categorized PCa patients into three immune subtypes and observed an obvious prevalence of *TP53* mutations, particularly the p.R248Q variant, within the immunosuppressed and nonimmune/immuneless subgroups. Interestingly, this variant was entirely absent in the immune-activated phenotype. These findings indicate a potential link between this mutation and the development of an immune-evasive tumor microenvironment.

### 3.2 CRISPR/Cas9 gene editing-enabled functional exploration of the *Trp53* p.R245Q point mutation in a mouse PCa model

**Figure 2:**
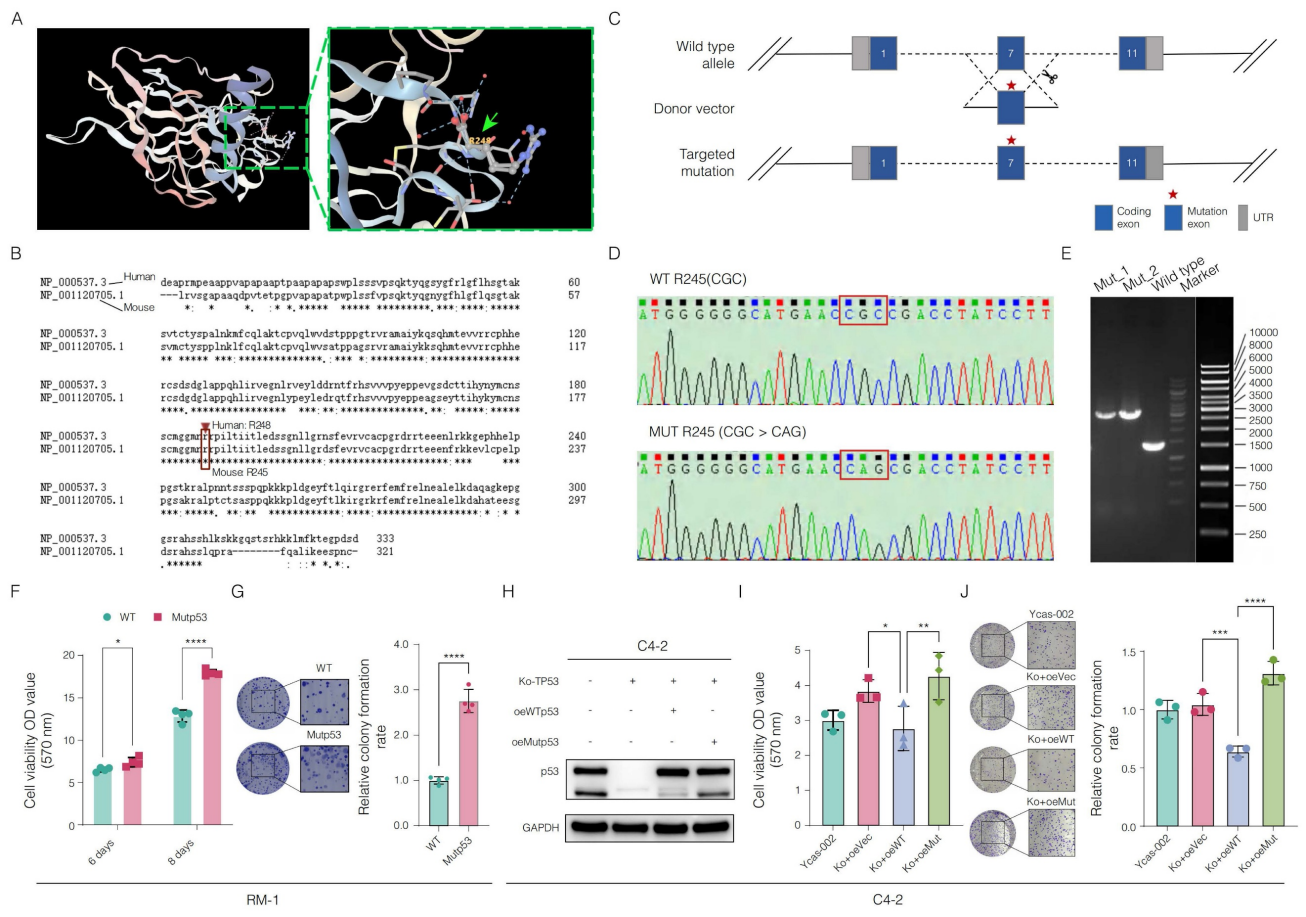
** Deciphering the impact of Mutp53 in prostate cancer (PCa) via CRISPR/Cas9 gene editing. A.** Illustration of the p53 protein structure highlighting the p.R248Q amino acid site, marked with a green arrow to emphasize its significance. **B.** Comparison of human and mouse *TP53 (Trp53)* coding amino acid sequences, illustrating genetic engineering techniques with a focus on the introduction of the mouse-equivalent R245Q mutation, corresponding to the human R248Q mutation. **C.** Detailed schematic of the CRISPR/Cas9 gene editing process, from the wild-type allele through the donor vector to the targeted mutation. **D-E.** Confirmation of the *Trp53*-R245Q mutation in RM-1 mouse cells via Sanger sequencing and gel electrophoresis. **F-G.** Functional assays showing increased cell proliferation (**F**) and colony formation (**G**) in RM-1 cells with the *Trp53*-R245Q mutation. **H.** Western blotting confirming the effective knockout of p53 and subsequent effects on the expression efficacy of wild-type (WTp53) and mutant p53 (mutp53) in human C4-2 PCa cells. Knocking out (ko) *TP53* in C4-2 cells via CRISPR/Cas9 technology. oe represents the reintroduction of wild-type *TP53* after knockout, whereas oe-Mutp53 represents the overexpression of the *TP53* p.R248Q mutant after knockout. The p53 protein bands were detected via a rabbit polyclonal antibody against p53 (Affinity, #AF0879). **I-J.** Comparative functional assays in C4-2 cells showing enhanced proliferation (**I**) and colony formation (**J**) with the *TP53*-R248Q mutation compared with the vector control and wild-type *TP53* sets. **Note:** To compare two groups, a t test was used. Comparisons among the four groups were conducted via one-way ANOVA. The values are mean ± SD of three independent experiments. Significance levels are represented as follows: *p < 0.05, **p < 0.01, ***p < 0.001, ****p < 0.0001.

We examined the structural features of the human p53 protein, with a particular focus on the p.R248Q amino acid site (**Figure 2A**). To investigate interactions between tumor and immune cells within the tumor microenvironment, we employed CRISPR/Cas9 gene editing to introduce the mouse-equivalent R245Q mutation-corresponding to the human p.R248Q variant, referred to as mutp53 (**Figure 2B**) into the RM-1 mouse PCa cell line. Successful integration of this mutation was verified through Sanger sequencing and gel electrophoresis following enzyme-specific digestion, as shown in **Figure 2C-E**. Functional assays of RM-1 cells revealed increases in both cell proliferation and colony formation capabilities due to this mutation, as demonstrated in **Figure 2F-G**.

To validate the effect of the *TP53* p.R248Q variant in a human PCa cell line, we knocked out the *TP53* gene in C4-2 cells, which naturally express wild-type p53. Reintroducing wild-type p53 markedly suppressed cancer cell growth and colony formation, which aligns with observations from previous studies 53. In contrast, introducing mutp53 resulted in marked increases in both cell growth and colony formation, similar to the effects observed in RM-1 cells (**Figure 2H-J**). These findings emphasize the critical role of the *TP53* p.R248Q variant in PCa progression and its potential as a therapeutic target.

### 3.3 Exploring the oncogenic function of Mutp53 in PCa progression

**Figure 3:**
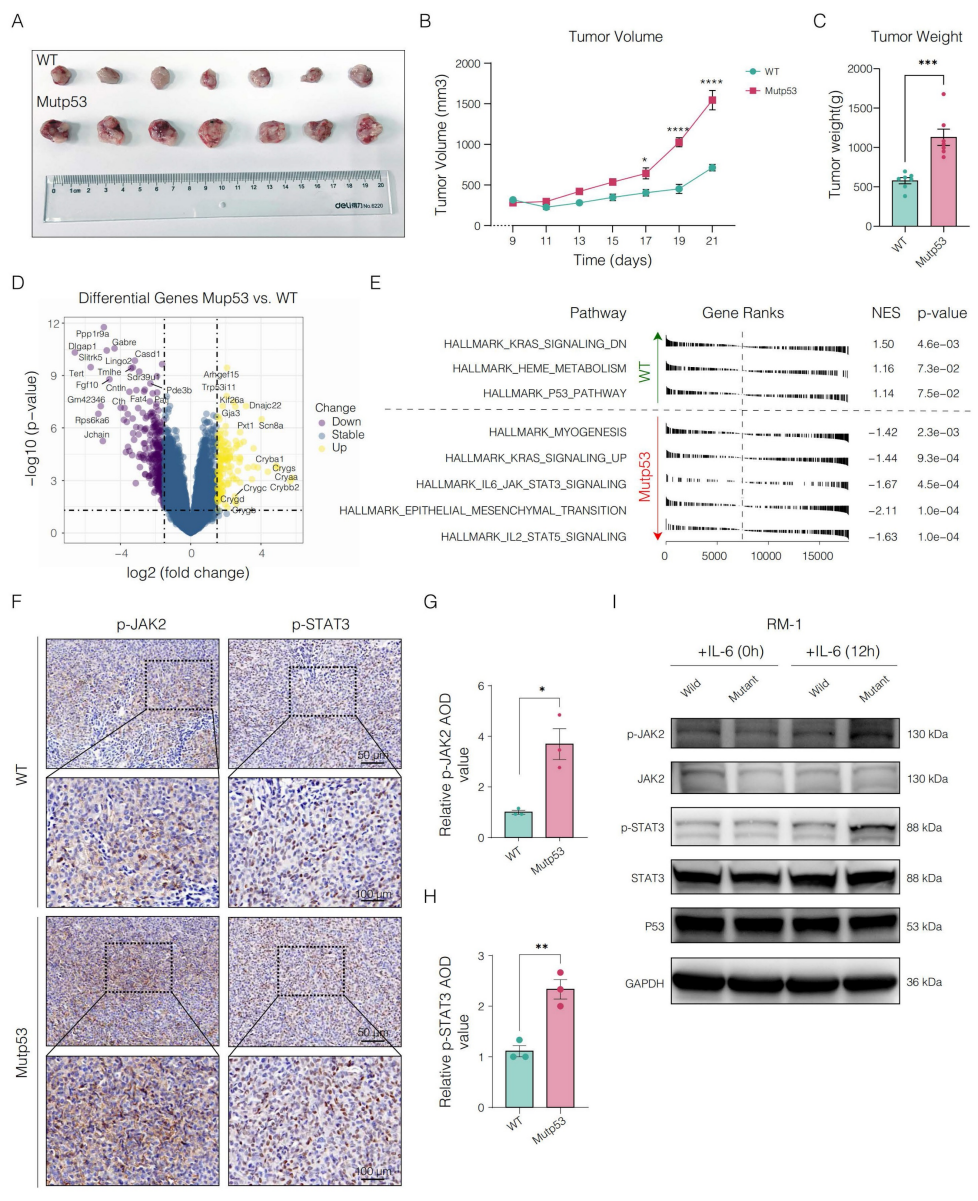
** Oncogenic potential and altered molecular signaling in Mutp53 prostate cancer (PCa). A.** Graphical representation comparing tumor sizes between mutant p53 (mutp53) and wild-type p53 (WTp53) mouse models (n = 7 per group). **B.** Time-course curves of tumor volume measurements for both the WTp53 and mutp53 groups (t test, p < 0.05). **C.** Comparative analysis of tumor weights between the mutp53 and WTp53 groups (t test, p < 0.001). **D.** Overview of genes differentially expressed in mutp53 versus WTp53 tumors. **E.** Hallmark pathway enrichment analysis of the differentially expressed genes revealed key signaling pathways altered in mutp53 tumors compared with WTp53 tumors. **F-H.** Immunohistochemical staining (**F**) and quantification of p-JAK2 (**G**) and p-STAT3 (**H**) expression in tumor tissues between the p53-mutated and p53-nonmutated groups. **I.** Western blotting assays showing increased levels of phosphorylated JAK2 and STAT3 in mutp53 PCa cells following IL-6 stimulation. **Note:** To compare two groups, a t test was used. The values are mean ± SD of three independent experiments. Significance levels are represented as follows: *p < 0.05, **p < 0.01, ***p < 0.001, ****p < 0.0001.

To investigate the role of mutp53 in PCa, we performed subcutaneous tumor implantation experiments in C57BL/6 mice. *In vivo* analysis revealed that tumors in the mutp53 group were significantly larger and heavier than those in the wild-type p53 (WTp53) group were, highlighting the increased tumorigenic capacity of mutp53-bearing cells (**Figure 3A-C**).

Subsequent RNA sequencing of samples from both groups helped identify critical genes or pathways potentially activated during tumor progression. The expression of key genes, including *ARHGEF15*, *KIF26A*, *DNAJC22*, *GJA3*, and *SCN8A*, was significantly upregulated in the mutp53 group, indicating enhanced tumor proliferation, invasion, migration, and antiapoptotic activities; conversely, the downregulation of genes such as *PPP1R9A*, *DLGAP1*, *GABRE*, *CASD1*, and *LINGO2* in the same group suggested disruptions in synaptic signaling, cellular architecture, and apoptosis in tumors expressing mutp53 (**Figure 3D** and **Supplementary Table 6**).

Pathway enrichment analysis revealed that mutations triggered the activation of several oncogenic pathways, notably KRAS signaling, IL6/JAK2/STAT3 signaling, and epithelial-mesenchymal transition (EMT) (**Figure 3E**). Given the pivotal role of the IL6/JAK2/STAT3 axis in promoting tumor progression and shaping an immunosuppressive microenvironment, we performed immunohistochemical analysis of phosphorylated JAK2 and STAT3 in tumor tissues from the mouse model to validate pathway activation. This analysis revealed a significant increase in staining scores in tumors harboring mutp53 (**Figure 3F**). Western blotting further validated these findings, demonstrating elevated levels of phosphorylated JAK2 and STAT3 in mutp53 cells following IL-6 stimulation, thereby confirming the mutation's role in activating the STAT3 signaling pathway. The data in Figure 3 and the supplementary materials demonstrate the oncogenic role of mutp53 in PCa, which is facilitated by the activation of the JAK2/STAT3 signaling pathway.

### 3.4 Mutp53 fosters an immunosuppressive TME in PCa

**Figure 4:**
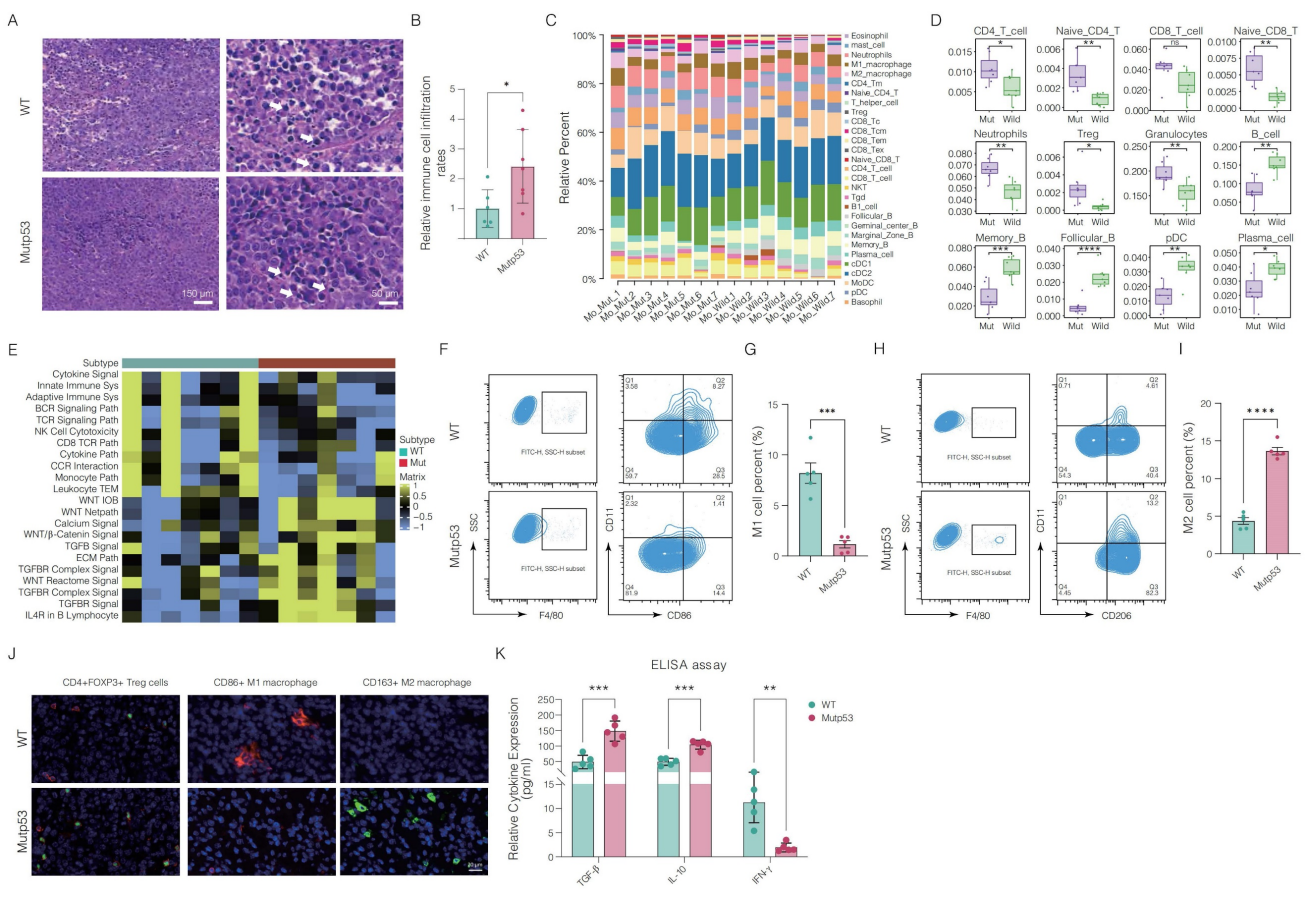
** Immunosuppressive tumor microenvironment in prostate tumors with Mutp53. A.** Histopathological comparison of lymphocyte infiltration in PCa tumors via H&E staining between mutp53 and WTp53 tumors (t test, p < 0.05). **B.** Quantitative analysis of H&E staining to assess lymphocyte density (t test, p < 0.05). **C.** Quantitative evaluation of immune cell infiltration within murine tumorigenic tissues via the seq-ImmuCC platform. **D.** Detailed quantification of differential immunocyte infiltration across tumor types. **E.** Analysis of immune-related pathways was performed by comparing WTp53 and mutp53 tumors to identify activated or suppressed signaling pathways. **F-I.** Flow cytometry analyses and quantification of immune cell subsets: F4/80+CD86+ M1 macrophages are shown in panel **F** with quantification in panel** G**, and F4/80+CD206+ M2 macrophages are shown in panel **H** with quantification in panel **I**. These panels compare the proportions of each cell type in tumors from both the WTp53 and mutp53 groups (t test, p = 0.002 and p < 0.001, respectively). **J.** Immunofluorescence staining showing the distribution of CD4+Foxp3+ Treg cells and macrophage phenotypes (CD86+M1 and CD163+M2) within tumors from the WTp53 and mutp53 groups. **K.** Enzyme-linked immunosorbent assay (ELISA) was used to measure the levels of the cytokines IFN-γ, IL-10, and TGF-β in mutp53 tumors compared with those in WTp53 tumors. **Note:** T tests were employed for comparisons between two groups, whereas one-way ANOVA was used for comparisons among the four groups. The values are mean ± SD of three independent experiments. Significance levels are denoted as *p < 0.05, **p < 0.01, ***p < 0.001, and ****p < 0.0001.

To investigate the immunological impact of mutp53 on PCa, we initially conducted a histopathological examination of the TME via H&E staining. This assessment revealed significantly greater lymphocyte infiltration in mutant tumors than in wild-type tumors (**Figure 4A-B**). Furthermore, we analyzed the infiltration of immune cells within murine tumorigenic tissues via the seq-ImmuCC platform (**Figure 4C**). **Figure 4D** shows that the mutp53 group exhibited greater infiltration of CD4+ T cells, neutrophils, Tregs, and granulocytes, whereas the infiltration of B cells, memory B cells, plasmacytoid dendritic cells (pDCs), and plasma cells decreased. Pathway enrichment analysis revealed a decrease in signaling activities related to cytokine interactions, the immune system's innate and adaptive components, B-cell receptor signaling, the CD8 TCR pathway, the monocyte pathway, and leukocyte transendothelial migration in the mutant groups. In contrast, activation of the WNT-beta-catenin, TGF-beta signaling, and extracellular matrix (ECM) pathways was noted (**Figure 4E**), indicating the establishment of an immunosuppressive microenvironment in PCa tumors with mutp53 mutations.

Moreover, flow cytometry analysis demonstrated a significant reduction in proinflammatory M1 macrophages and an increase in tumor-promoting M2 macrophages in mutp53 tumors compared to WTp53 tumors, indicating a shift in macrophage function (**Figure 4F-I**). Immunofluorescence assays corroborated these findings, revealing a reduced percentage of CD86+ M1 macrophages and an increased percentage of CD163+ M2 macrophages and CD4+Foxp3+ Treg cells in mutated tumor tissues (**Figure 4J**). Additionally, the levels of proinflammatory cytokines, including IFN-γ, IL-10, and TGF-β, were significantly lower in mutp53 tumors than in WTp53 tumors (**Figure 4K**). Collectively, these findings indicate that mutp53 promotes an immunosuppressive microenvironment, which may contribute to tumor growth.

### 3.5 Elucidating the mechanism of STAT3 activation by Mutp53 via SHP1 interaction

**Figure 5:**
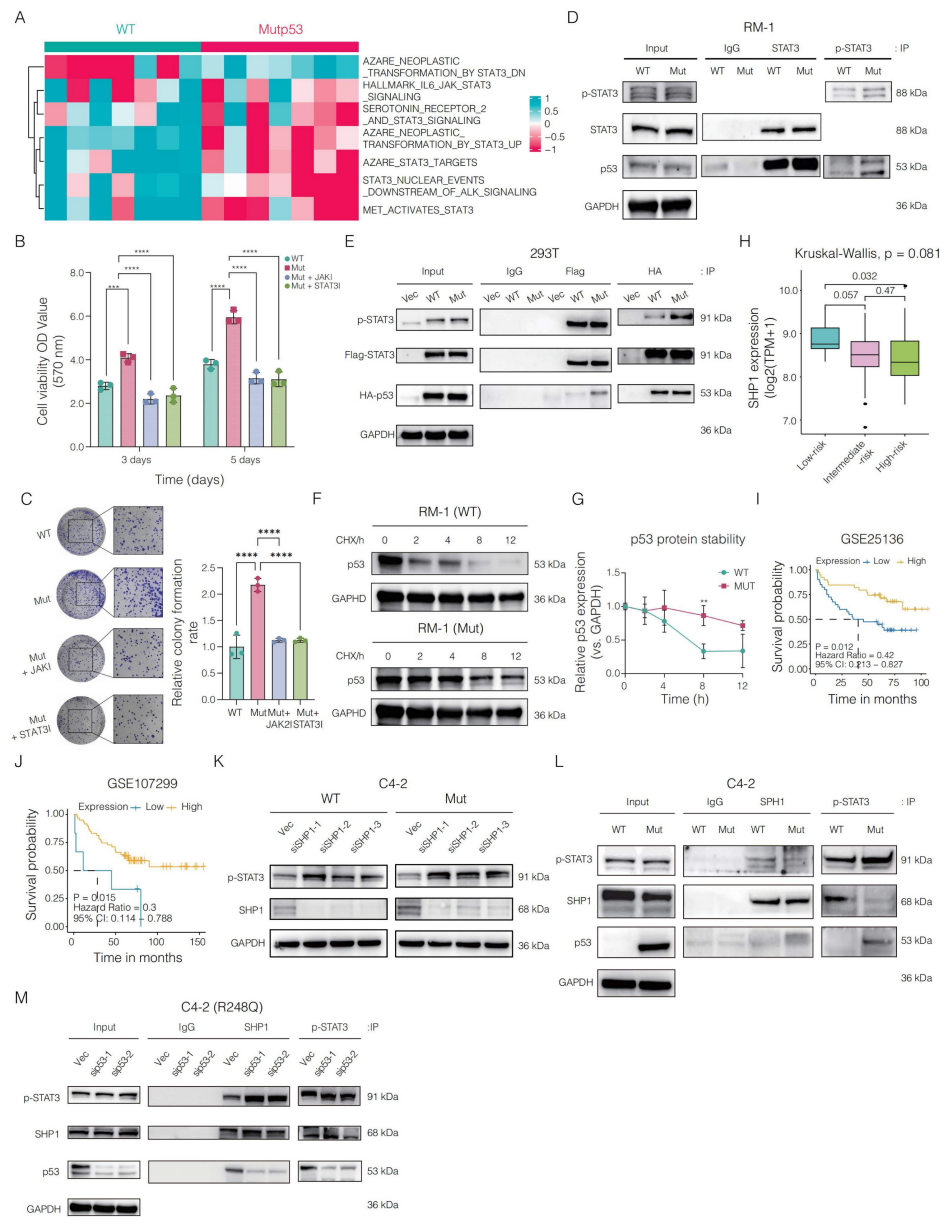
** Impact of Mutp53 on JAK2/STAT3 signaling activation in prostate cancer (PCa). A.** Heatmap illustrating the differentially activated pathways associated with JAK2 and STAT3 signaling in mutp53 versus WTp53 tumors. **B-C.** Assays for cell viability (**B**) and colony formation (**C**) comparing the proliferation of WTp53 and mutp53 RM-1 cells treated with JAK2 and STAT3 inhibitors. **D-E.** Coimmunoprecipitation assay demonstrating variations in the binding of the STAT3 and p-STAT3 proteins to mutp53 compared with WTp53 in RM-1 cells (edited via CRISPR/Cas9) and 293T cells (expressing ectopic flag-STAT3 along with HA-tagged WTp53 and Mutp53, respectively). **F-G.** Western blotting (**F**) and quantification (**G**) showing differences in protein stability between mutp53 and WTp53 in RM-1 cells treated with cycloheximide (CHX, a protein synthesis inhibitor). **H.** Kruskal‒Wallis test comparing *SHP1* expression across low-risk, intermediate-risk, and high-risk PCa patients. **I‒J.** Survival analyses correlating *SHP1* expression with recurrence-free survival in PCa patients derived from the GSE25136 and GSE107299 cohorts (GSE25136: p = 0.012, HR = 0.42, 95% CI=0.213‒0.827; GSE107299: p = 0.012, HR = 0.42, 95% CI=0.213‒0.827). **K.** Western blotting assay demonstrating the efficacy of small interfering RNAs targeting *SHP1* in KO-*TP53* C4-2 cells expressing WTp53 or mutp53 and interference with the expression of p-STAT3. **L.** Coimmunoprecipitation showing variations in the binding between SHP1 and p-STAT3 in *TP53*-KO C4-2 cells expressing either vector or mutp53, with immunoprecipitation targeting SHP1 and p-STAT3. **M.** Coimmunoprecipitation showing changes in SHP1 and p-STAT3 binding after mutp53 expression was reduced in *TP53*-KO C4-2 cells re-expressing mutp53, and SHP1 and p-STAT3 were immunoprecipitated. **Note:** T tests were employed for comparisons between two groups, whereas one-way ANOVA was used for comparisons among the four groups. The values are mean ± SD of three independent experiments. Significance levels are denoted as *p < 0.05, **p < 0.01, ***p < 0.001, and ****p < 0.0001.

We analyzed a collection of pathways associated with JAK/STAT signaling and, on the basis of the TCGA-PRAD cohort, observed that the activity of KEGG_JAK_STAT_SIGNALING_PATHWAY was greater in samples with the p.R248Q mutation than in those with WTp53 or other *TP53* mutations (**Supplementary Figure 1**). To further explore the oncogenic role of mutp53 in activating the STAT3 signaling pathway in PCa, we systematically analyzed genes associated with or regulated by this pathway and found that most of these genes were activated following mutation (**Figure 5A**). Subsequent *in vitro* experiments involved treating RM-1 cells with JAK2/STAT3 pathway inhibitors. Cell proliferation was assessed via the MTT assay, which revealed a significant decrease in the proliferation of mutp53 cells following treatment (**Figure 5B**). Additionally, cell colony formation assays indicated that the use of these inhibitors markedly reversed the ability of the mutp53 tumor cells to form colonies (as illustrated in **Figure 5C**).

Mutp53 impacts p53 function through modifications in protein structure, DNA binding, transcriptional activity, and stability. We hypothesized that the activation of STAT3 by mutp53 occurs via physical interactions. Coimmunoprecipitation revealed increased binding of both STAT3 and p-STAT3 to mutp53 compared with that in WTp53 cells (**Figure 5D**). This finding was corroborated in human 293T cells ectopically expressing wild-type HA-tagged TP53, mutant HA-tagged TP53, or FLAG-tagged STAT3, where the interaction between STAT3/p-STAT3 and mutp53 was stronger than that between STAT3 and WTp53 (**Figure 5E**). Additionally, we analyzed the degradation kinetics of p53 following mutation. Western blotting via a CHX chase assay revealed that mutp53 is more stable than WTp53 (**Figure 5F-G**).

We next investigated the mechanism by which mutp53 enhances STAT3 signaling, focusing on its effect on STAT3 phosphorylation levels. Since SHP1 and SHP2, both protein tyrosine phosphatases, are known to negatively regulate STAT3 via dephosphorylation, we assessed their roles in PCa. Our analysis identified *SHP1* as a protective factor, supporting the tumor-suppressive implications highlighted in our study. Analysis of the GSE25136 and GSE107299 datasets revealed that elevated *SHP1* expression correlated with improved recurrence-free survival (RFS) in PCa patients (**Figure 5H-I**). Furthermore, Kruskal-Wallis analysis showed that individuals in the low-risk category exhibited higher median *SHP1* levels compared to those at high risk (**Figure 5J**). In addition, we assessed the association between *SHP1* expression and key antitumor cytokines. Patients with higher *SHP1* levels demonstrated significantly increased expression of immune mediators such as *IL-12B*, *TNF*, *CXCL10*, *IFNB1*, *HLA-DRA*, and *CCL5*, compared to the low *SHP1* group (**Supplementary Figure 2**), suggesting a potential link between *SHP1* expression and enhanced antitumor immunity.

In-depth mechanistic studies revealed that knocking down the *SHP1* gene in *TP53*-KO C4-2 cells expressing either WTp53 or mutp53 resulted in the upregulation of p-STAT3 (**Figure 5K**). We then ectopically expressed the vector and mutp53 in *TP53*-KO C4-2 cells. Coimmunoprecipitation assays indicated that SHP1 could bind to p-STAT3, but mutp53 disrupted this interaction by competitively binding with p-STAT3, thereby sustaining STAT3 phosphorylation (**Figure 5L**). To further validate these findings, we silenced mutp53 expression in *TP53*-KO C4-2 cells reexpressing mutp53. Subsequent coimmunoprecipitation assays targeting SHP1 and p-STAT3 demonstrated that reducing mutp53 expression increased the interaction between SHP1 and p-STAT3 (**Figure 5M**). Collectively, these results indicate that mutp53 enhances STAT3 signaling activation in PCa by competitively binding to p-STAT3, inhibiting its dephosphorylation by SHP1 and thus promoting oncogenic processes.

### 3.6 Preclinical assessment of JAK2 and STAT3 blockade in controlling Mutp53-driven CRPC

**Figure 6:**
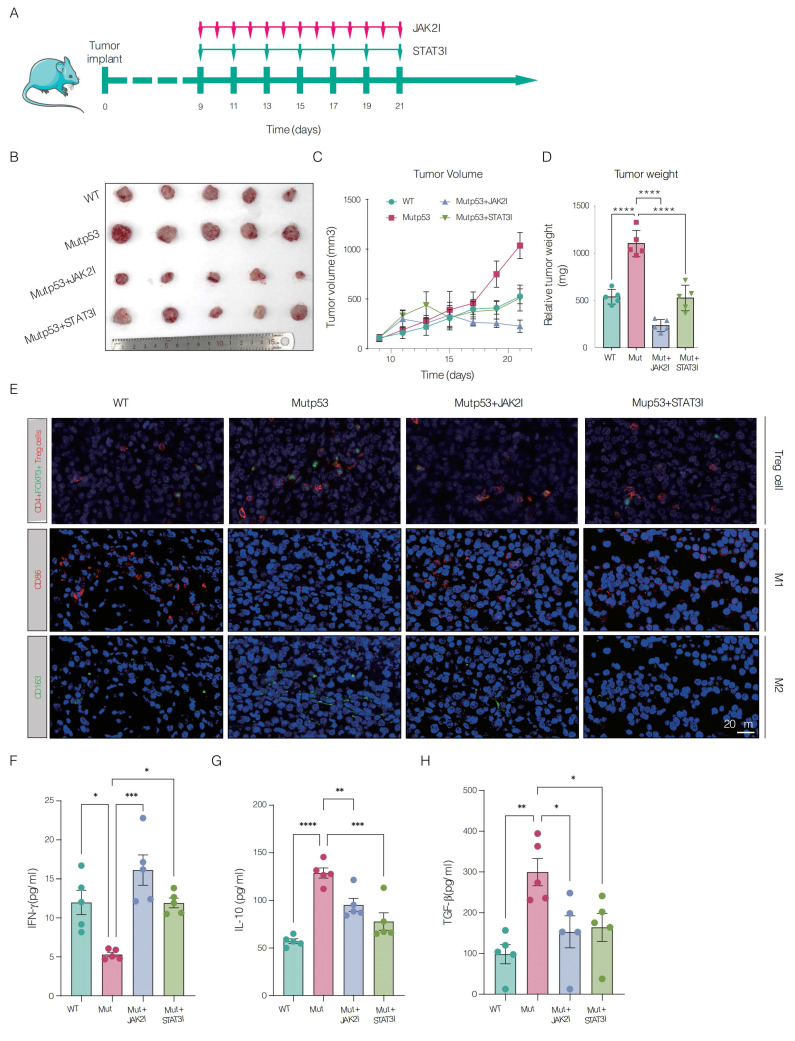
** Preclinical assessment of JAK2 and STAT3 blockade in controlling Mutp53-driven CRPC.** A. Schematic illustrating the treatment timeline for mice with wild-type p53 (WTp53) and mutant p53 (mutp53) tumors treated with JAK2 and STAT3 inhibitors, respectively (n = 5 per group). **B.** Graphical representation comparing tumor sizes between the WTp53 and mutp53 mouse models, including those treated with JAK2 and STAT3 inhibitors. **C.** Time‒course curves depicting tumor volume measurements for mouse models expressing WTp53, mutp53, and mutp53 treated with JAK2 and STAT3 inhibitors. **D.** Comparative analysis of tumor weights across mouse models expressing WTp53, mutp53, or mutp53 and treated with JAK2 or STAT3 inhibitors. **E.** Immunofluorescence staining showing the infiltration of CD4+Foxp3+ Treg cells, CD86^+^ M1 macrophages, and CD163^+^ M2 macrophages in WTp53, mutp53, and mutp53 model mice treated with JAK2 or STAT3 inhibitors, respectively. **F-H.** Enzyme-linked immunosorbent assays (ELISAs) showing the levels of the cytokines IFN-γ, IL-10, and TGF-β in different mouse models, including the WTp53, mutp53, and mutp53 mouse models treated with JAK2 or STAT3 inhibitors. **Note:** T tests were employed for comparisons between two groups, whereas one-way ANOVA was used for comparisons among the four groups. The values are mean ± SD of three independent experiments. Significance levels are indicated as *p < 0.05, **p < 0.01, ***p < 0.001, and ****p < 0.0001.

In our preclinical *in vivo* mouse model, we investigated the therapeutic potential of inhibiting JAK2 and STAT3 signaling to mitigate CRPC progression driven by Mutp53. Mice bearing mutp53 tumors received treatment with inhibitors specifically targeting the JAK2 and STAT3 pathways. The treatment led to a substantial reduction in tumor weight and volume, as shown in **Figure 6A-D**. Immunofluorescence assays confirmed that JAK2 and STAT3 inhibition increased the infiltration of CD4+Foxp3+ Treg cells and CD86+ M1 macrophages while decreasing the proportion of CD163+ M2 macrophages, as shown in **Figure 6E**. Additionally, ELISA demonstrated that IFN-γ levels were significantly greater in the posttreatment group than in the mutp53 group, as depicted in **Figure 6F**. Moreover, the levels of the cytokines IL-10 and TGF-β, which are elevated in mutant tumors, were significantly reduced after treatment, as detailed in **Figure 6G-H**. Collectively, these findings validate the efficacy of JAK2 and STAT3 inhibitors in suppressing tumor growth and modulating the immune landscape in a mouse model of mutp53-driven CRPC.

## 4. Discussion

Here, we systematically demonstrated that *TP53* gene mutations, particularly the p.R248Q variant, profoundly affect the progression of PCa by promoting an immunosuppressive TME. Furthermore, the use of JAK2 and STAT3 inhibitors has shown promising results in reversing these effects, significantly reducing the tumor burden and altering the immune landscape in a mutp53-driven CRPC mouse model. These findings are particularly significant because they align with emerging evidence suggesting that dysregulation of JAK/STAT signaling is a key factor in the immune suppression observed in various solid tumors, including PCa. As noted in previous studies of the TME, IL-6 trans-signaling promotes the interaction between GP130, soluble IL-6 receptor (sIL-6R), and IL-6, driving JAK-STAT-dependent tumor cell migration. This signaling pathway also recruits neutrophils and macrophages via MCP-1 secretion, promotes M2 macrophage polarization, inhibits dendritic cell activation, and enhances Treg differentiation, contributing to an immunosuppressive TME that supports tumor progression 54-58.

In our study, we observed that *TP53* mutations are highly prevalent in PCa patients and are closely associated with an aggressive cancer phenotype characterized by poor survival and a high recurrence rate, which is consistent with pivotal prior studies. For example, Matthew P. Deek reported that patients harboring *TP53* mutations exhibited a progression-free survival (PFS) that was six months shorter compared to those without such mutations. Donehower *et al.*'s analysis of data from The Cancer Genome Atlas (TCGA) identified *TP53* as a critical mutation across multiple cancers, emphasizing its central role in cancer progression. These findings underscore the pivotal role of *TP53* mutations in PCa and reinforce the necessity of targeting them for the advancement of effective therapeutic strategies 59-63. Notably, we documented a correlation between mutp53 and the immunosuppressive or nonimmune or nonimmune microenvironment, further supporting this theory, which was corroborated by recent immunogenomic analyses by Rooney *et al.* 64 and our center 33. Our previous study 33 on PCa highlighted the role of immune responses in shaping clinical outcomes, particularly the impact of immunosuppressive and immunoactive environments on patient prognosis. This finding aligns with earlier findings by Rooney *et al.*, who reported immune cytolytic activity and key immune evasion mechanisms across multiple tumor types. Both studies underscore the importance of tumor-immune interactions, especially in modulating treatment responses. Our current work further elucidates how specific mutations, such as *TP53*, influence the immunosuppressive TME and highlights the potential for targeted therapies such as JAK2/STAT3 inhibitors in CRPC. To further elucidate the effects of mutp53 on the immune microenvironment, we utilized CRISPR/Cas9 technology to edit the RM-1 mouse PCa cell line genetically, introducing the p.R245Q mutation corresponding to the human p.R248Q mutation. Conducting tumor experiments in mice with normal immune functions allowed us not only to observe the immune response to tumor progression and the interplay between the tumor and the immune system but also to validate the results through a series of experiments, including RNA sequencing of immune cell components, flow cytometry, and immunofluorescence assays, which revealed that mutp53 orchestrates the formation of a suppressive tumor immune microenvironment. This environment significantly promotes tumor progression and metastasis 65-67. In this research, the application of CRISPR/Cas9 technology proved to be highly important. Through precise genetic editing and permanent gene modification, this technique enables us to study the expression and regulation of specific mutations under nearly physiological conditions 68, 69. We systematically investigated tumor cell dynamics in immunocompetent mice harboring the *Trp53* p.R245Q mutation, with emphasis on how these mutant cells interact with the immune system. Compared to WTp53 tumors, mutant epithelial cancer cells exhibited distinct communication patterns with CD86+ M1 macrophages, CD163+ M2 macrophages, and CD4+Foxp3+ regulatory T cells, fostering an immunosuppressive tumor microenvironment. In addition, ELISA assays revealed elevated concentrations of IL-10 and TGF-β, both key immunosuppressive cytokines, alongside a marked decrease in the proinflammatory mediator IL-1β in Mutp53 tumors. These results enhance our understanding of how this *TP53* mutation shapes immune responses within tumors and inform the development of precision immunotherapies.

Our analysis revealed significant activation of JAK/STAT3, KRAS signaling, epithelial-EMT, and IL2/STAT5 signaling in the Mutp53 group, highlighting the broad impact of the p.R248Q mutation in PCa. Mutp53 cooperates with KRAS mutations to enhance tumor progression by activating CREB1, which upregulates FOXA1 and WNT/β-catenin, promoting metastasis 70. Additionally, mutp53 maintains KRAS activity by regulating GTPase-activating proteins (GAPs), further supporting its oncogenic effects 71. Mutp53 also promotes EMT through factors such as ZEB1 and SNAIL, which drive invasion, and modulates IL2/STAT5 signaling, contributing to immune evasion and tumor progression. These findings underscore the role of p.R248Q *TP53* mutations in driving PCa biology and highlight potential therapeutic targets for these interconnected pathways 72-74. STAT3 signaling is crucial for cell proliferation, survival, and immune evasion in PCa. 75-77, significantly contributes to a tumor-permissive environment 57, 78, 79. Its abnormal activation is driven by factors such as mutations in receptors, kinases, and transcription factors. Mutp53 influences cytokine secretion, modulating levels of IL-6, IL-10, and IL-11 71, 80, 81. Additionally, Mutp53 may engage with signaling pathways such as NF-κB and PI3K/AKT, amplifying JAK/STAT activation and driving tumor progression 82-84. The TME also plays a key role in JAK/STAT activation. Cytokines from immune cells and signals from tumor-associated fibroblasts (CAFs) increase pathway activity 85. External factors, such as oxidative stress and ECM interactions, also modulate STAT3 signaling 86, 87. Mutations in JAK2 or STAT3 and the loss of negative regulators such as SHP2 and SOCS further sustain this activation 88, 89. Our study revealed how the p.R248Q *TP53* mutation disrupts the SHP1/STAT3 interaction, leading to sustained STAT3 activation. These findings underscore the role of* TP53* mutations in driving JAK/STAT activation in CRPC, providing deeper insight into the regulatory network governing JAK/STAT signaling in PCa. SHP1 influences tyrosine phosphorylation-mediated cellular processes and is overexpressed in PCa cells 90-92, serving as a prognostic marker for survival following radical prostatectomy 93. Notably, overexpressing SHP1 in ALK^+^ anaplastic large-cell lymphoma cells has been shown to reverse JAK3 and STAT3 activation, reducing STAT3 target expression 94. SHP1 is crucial in modulating JAK/STAT signaling, thereby enhancing the effectiveness of chemotherapeutic agents. For example, guggulsterone, a phytosteroid, curbs cancer cell proliferation by inducing SHP1, which inhibits JAK2 and STAT3 phosphorylation 95. Similarly, dovitinib activates SHP1, diminishing STAT3 activity and augmenting apoptosis in hepatocellular carcinoma 96, suggesting its synergistic potential with sorafenib to combat chemoresistance. Plumbagin, derived from medicinal plants, induces SHP1 in multiple myeloma cells, reducing STAT3 phosphorylation 97. Although the role of SHP1 in PCa has been reviewed, the detailed mechanisms by which SHP1 regulates STAT3 activation in the presence of Mutp53 remain underexplored. Our study demonstrated that mutp53 interferes with the SHP1-STAT3 interaction, leading to persistent STAT3 activation and accelerated CRPC progression. Notably, administering JAK2 and STAT3 pathway inhibitors in our mouse models reduced tumor growth and altered the immune landscape, suggesting compelling therapeutic avenues. Upon treatment with JAK2/STAT3 inhibitors, we observed a reversal of these immunosuppressive conditions. Specifically, the proportion of M2 macrophages decreased, whereas that of M1 macrophages increased. Correspondingly, the levels of IL-10 and TGF-β were reduced, whereas the level of IL-1β was increased. These findings suggest that targeting the SHP1/STAT3 pathway could effectively counteract the mutp53-driven immunosuppressive TME, underscoring its therapeutic potential in CRPC treatment. These findings align with those of previous studies 98. This review details the diverse functions of STAT3 in oncogenesis, further supporting our results that demonstrate the therapeutic potential of targeting STAT3 in mutp53-driven CRPC. Our study highlights the significant role that *TP53* mutations, particularly the p.R248Q variant, play in promoting an immunosuppressive TME in PCa. These findings suggest that the *TP53* mutation status could serve as a valuable biomarker for identifying patients who may benefit from specific therapeutic approaches. For example, patients with *TP53* mutations may be more responsive to therapies that target the immunosuppressive pathways driven by these mutations, such as JAK2/STAT3 inhibitors. Additionally, understanding the specific *TP53* mutation profile in PCa patients could guide the selection of immunotherapeutic strategies. For example, patients harboring the p.R248Q mutation, which is associated with a more aggressive and immunosuppressive tumor phenotype, might benefit from combination therapies that include immune checkpoint inhibitors alongside treatments targeting *TP53*-mediated pathways. By integrating *TP53* mutation analysis into clinical decision-making, it may be possible to personalize treatment plans and improve outcomes for PCa patients. We believe that these expanded considerations significantly enhance the relevance and applicability of our research to clinical practice. Our study highlights the crucial role of *TP53* mutations, particularly the p.R248Q variant, in shaping the tumor immune microenvironment. By disrupting the SHP1/STAT3 interaction, *TP53* mutations sustain STAT3 activation, fostering an immunosuppressive environment with increased M2 macrophages, decreased M1 macrophages, and altered cytokine profiles. These findings reveal a key pathway through which *TP53* mutations drive immune evasion and tumor progression. Future research could focus on developing therapies that inhibit the SHP1/STAT3 pathway or combine this approach with strategies targeting the immunosuppressive microenvironment, potentially leading to more effective treatments for castration-resistant PCa.

Our study underscores the role of Mutp53 in STAT3 activation and immune suppression in CRPC, yet several limitations should be acknowledged. We focused on the p.R248Q mutation due to its high prevalence and well-established association with immunosuppression. However, other *TP53* hotspot mutations (e.g., p.R175H and p.R273C), as well as frequently altered genes such as *PTEN* and *MYC*, may have cooperative effects that warrant further investigation. While our work provides valuable insights into SHP1/STAT3 signaling in PCa, the inherent differences between mouse models and human biology must be considered. Mouse models, though indispensable for studying cancer progression and immune responses, do not fully capture the complexity of the human immune system and TME. Variations in immune cell composition and cytokine signaling, for instance, may influence the clinical relevance of our findings. To address these challenges, we carefully considered such differences in our experimental design and plan to further validate our results using human-derived models and clinical samples. This strategy will help ensure the translational potential of our findings and support their application in developing effective therapies for PCa patients.

In conclusion, this study highlights the pivotal role of *TP53* mutations, particularly the p.R248Q variant, in promoting CRPC progression and fostering tumor immune suppression. By demonstrating the effectiveness of JAK2 and STAT3 inhibitors in preclinical models, our findings support their potential as therapeutic strategies to counteract mutp53-induced tumor aggressiveness and immunosuppression, paving the way for targeted therapies that could significantly improve outcomes in CRPC treatment.

## Supplementary Material

Supplementary figures and tables.

## Abbreviations

PFS: progression-free survival
CRPC: castration-resistant prostate cancer
PCa: prostate cancer
mutp53: p53 missense mutants
GOF: gain-of-function
FBS: fetal bovine serum
Co-IP: Coimmunoprecipitation
MTT: 3-(4,5-dimethyl-2-thiazolyl)-2,5-diphenyl-2-H-tetrazolium bromide
OD: optical density
H&E: hematoxylin and eosin staining
IHC: Immunohistochemistry
ELISA: Enzyme-linked immunosorbent assay
WTp53: wild-type p53
EMT: epithelial-mesenchymal transition
pDCs: plasmacytoid dendritic cells
ECM: extracellular matrix
SHP1: tyrosine phosphatases SH2 domain-containing phosphatase-1
TME: tumor microenvironment
sIL-6R: soluble IL-6 receptor
TCGA: The Cancer Genome Atlas
GAPs: GTPase-activating proteins
CAFs: tumor-associated fibroblasts

## References

[1] Wu Z, Xia F, Lin R (2024). Global burden of cancer and associated risk factors in 204 countries and territories, 1980-2021: a systematic analysis for the GBD 2021. Journal of hematology & oncology.

[2] Xuan Z, Chen C, Sun H, Yang K, Li J, Fu M (2024). NDR1/FBXO11 promotes phosphorylation-mediated ubiquitination of β-catenin to suppress metastasis in prostate cancer. Int J Biol Sci.

[3] Liu Y, Chen S, Guo K, Ma S, Wang X, Liu Q (2024). Osteoblast-derived exosomal miR-140-3p targets ACER2 and increases the progression of prostate cancer via the AKT/mTOR pathway-mediated inhibition of autophagy. Faseb j.

[4] Bergengren O, Pekala KR, Matsoukas K, Fainberg J, Mungovan SF, Bratt O (2023). 2022 Update on Prostate Cancer Epidemiology and Risk Factors-A Systematic Review. European urology.

[5] Rebello RJ, Oing C, Knudsen KE, Loeb S, Johnson DC, Reiter RE (2021). Prostate cancer. Nature reviews Disease primers.

[6] Porkka KP, Visakorpi T (2004). Molecular mechanisms of prostate cancer. European urology.

[7] Knowell AE, Patel D, Morton DJ, Sharma P, Glymph S, Chaudhary J (2013). Id4 dependent acetylation restores mutant-p53 transcriptional activity. Molecular cancer.

[8] Ding D, Blee AM, Zhang J, Pan Y, Becker NA, Maher LJ 3rd (2023). Gain-of-function mutant p53 together with ERG proto-oncogene drive prostate cancer by beta-catenin activation and pyrimidine synthesis. Nature communications.

[9] Zhao Y, Ding L, Wang D, Ye Z, He Y, Ma L (2019). EZH2 cooperates with gain-of-function p53 mutants to promote cancer growth and metastasis. The EMBO journal.

[10] Valentino E, Bellazzo A, Di Minin G, Sicari D, Apollonio M, Scognamiglio G (2017). Mutant p53 potentiates the oncogenic effects of insulin by inhibiting the tumor suppressor DAB2IP. Proceedings of the National Academy of Sciences of the United States of America.

[11] Kastenhuber ER, Lowe SW (2017). Putting p53 in Context. Cell.

[12] Muller PA, Vousden KH (2014). Mutant p53 in cancer: new functions and therapeutic opportunities. Cancer Cell.

[13] Lawrence MS, Stojanov P, Mermel CH, Robinson JT, Garraway LA, Golub TR (2014). Discovery and saturation analysis of cancer genes across 21 tumour types. Nature.

[14] Sigal A, Rotter V (2000). Oncogenic mutations of the p53 tumor suppressor: the demons of the guardian of the genome. Cancer research.

[15] Lang GA, Iwakuma T, Suh YA, Liu G, Rao VA, Parant JM (2004). Gain of function of a p53 hot spot mutation in a mouse model of Li-Fraumeni syndrome. Cell.

[16] Freed-Pastor WA, Mizuno H, Zhao X, Langerød A, Moon SH, Rodriguez-Barrueco R (2012). Mutant p53 disrupts mammary tissue architecture via the mevalonate pathway. Cell.

[17] Dittmer D, Pati S, Zambetti G, Chu S, Teresky AK, Moore M (1993). Gain of function mutations in p53. Nature genetics.

[18] Hanel W, Marchenko N, Xu S, Yu SX, Weng W, Moll U (2013). Two hot spot mutant p53 mouse models display differential gain of function in tumorigenesis. Cell Death Differ.

[19] Olive KP, Tuveson DA, Ruhe ZC, Yin B, Willis NA, Bronson RT (2004). Mutant p53 gain of function in two mouse models of Li-Fraumeni syndrome. Cell.

[20] Walerych D, Lisek K, Sommaggio R, Piazza S, Ciani Y, Dalla E (2016). Proteasome machinery is instrumental in a common gain-of-function program of the p53 missense mutants in cancer. Nature cell biology.

[21] Zhu J, Sammons MA, Donahue G, Dou Z, Vedadi M, Getlik M (2015). Gain-of-function p53 mutants co-opt chromatin pathways to drive cancer growth. Nature.

[22] Xu J, Reumers J, Couceiro JR, De Smet F, Gallardo R, Rudyak S (2011). Gain of function of mutant p53 by coaggregation with multiple tumor suppressors. Nature chemical biology.

[23] Do PM, Varanasi L, Fan S, Li C, Kubacka I, Newman V (2012). Mutant p53 cooperates with ETS2 to promote etoposide resistance. Genes & development.

[24] Di Agostino S, Strano S, Emiliozzi V, Zerbini V, Mottolese M, Sacchi A (2006). Gain of function of mutant p53: the mutant p53/NF-Y protein complex reveals an aberrant transcriptional mechanism of cell cycle regulation. Cancer Cell.

[25] Lee MK, Teoh WW, Phang BH, Tong WM, Wang ZQ, Sabapathy K (2012). Cell-type, dose, and mutation-type specificity dictate mutant p53 functions in vivo. Cancer Cell.

[26] de Vries A, Flores ER, Miranda B, Hsieh HM, van Oostrom CT, Sage J (2002). Targeted point mutations of p53 lead to dominant-negative inhibition of wild-type p53 function. Proceedings of the National Academy of Sciences of the United States of America.

[27] Hegi ME, Klein MA, Rüedi D, Chène P, Hamou MF, Aguzzi A (2000). p53 transdominance but no gain of function in mouse brain tumor model. Cancer research.

[28] Srivastava S, Wang S, Tong YA, Hao ZM, Chang EH (1993). Dominant negative effect of a germ-line mutant p53: a step fostering tumorigenesis. Cancer research.

[29] Boettcher S, Miller PG, Sharma R, McConkey M, Leventhal M, Krivtsov AV (2019). A dominant-negative effect drives selection of TP53 missense mutations in myeloid malignancies. Science (New York, NY).

[30] Liu H, Tang L, Li Y, Xie W, Zhang L, Tang H (2024). Nasopharyngeal carcinoma: current views on the tumor microenvironment's impact on drug resistance and clinical outcomes. Molecular cancer.

[31] Tang Y, Cai Q, Tian Z, Chen W, Tang H (2025). Crosstalk between Gut Microbiota and Cancer Immunotherapy: Present Investigations and Future Perspective. Research (Wash D C).

[32] Yang C, Deng X, Tang Y, Tang H, Xia C (2024). Natural products reverse cisplatin resistance in the hypoxic tumor microenvironment. Cancer Lett.

[33] Meng J, Zhou Y, Lu X, Bian Z, Chen Y, Zhou J (2021). Immune response drives outcomes in prostate cancer: implications for immunotherapy. Molecular oncology.

[34] Kang JG, Lago CU, Lee JE, Park JH, Donnelly MP, Starost MF (2020). A Mouse Homolog of a Human TP53 Germline Mutation Reveals a Lipolytic Activity of p53. Cell reports.

[35] van Boxtel R, Kuiper RV, Toonen PW, van Heesch S, Hermsen R, de Bruin A (2011). Homozygous and heterozygous p53 knockout rats develop metastasizing sarcomas with high frequency. Am J Pathol.

[36] Yu X, Zhang Y, Xiong S, McDaniel JM, Sun C, Chau GP (2022). Omics analyses of a somatic Trp53(R245W/+) breast cancer model identify cooperating driver events activating PI3K/AKT/mTOR signaling. Proceedings of the National Academy of Sciences of the United States of America.

[37] Ely ZA, Mathey-Andrews N, Naranjo S, Gould SI, Mercer KL, Newby GA (2024). A prime editor mouse to model a broad spectrum of somatic mutations in vivo. Nat Biotechnol.

[38] Murai K, Skrupskelyte G, Piedrafita G, Hall M, Kostiou V, Ong SH (2018). Epidermal Tissue Adapts to Restrain Progenitors Carrying Clonal p53 Mutations. Cell Stem Cell.

[39] Percie du Sert N, Hurst V, Ahluwalia A, Alam S, Avey MT, Baker M (2020). The ARRIVE guidelines 2.0: Updated guidelines for reporting animal research. PLoS Biol.

[40] de Bruijn I, Kundra R, Mastrogiacomo B, Tran TN, Sikina L, Mazor T (2023). Analysis and Visualization of Longitudinal Genomic and Clinical Data from the AACR Project GENIE Biopharma Collaborative in cBioPortal. Cancer research.

[41] Yang S, Giannone RJ, Dice L, Yang ZK, Engle NL, Tschaplinski TJ (2012). Clostridium thermocellum ATCC27405 transcriptomic, metabolomic and proteomic profiles after ethanol stress. BMC genomics.

[42] Sinha A, Huang V, Livingstone J, Wang J, Fox NS, Kurganovs N (2019). The Proteogenomic Landscape of Curable Prostate Cancer. Cancer Cell.

[43] Fraser M, Livingstone J, Wrana JL, Finelli A, He HH, van der Kwast T (2021). Somatic driver mutation prevalence in 1844 prostate cancers identifies ZNRF3 loss as a predictor of metastatic relapse. Nature communications.

[44] Bolger AM, Lohse M, Usadel B (2014). Trimmomatic: a flexible trimmer for Illumina sequence data. Bioinformatics (Oxford, England).

[45] Kim D, Paggi JM, Park C, Bennett C, Salzberg SL (2019). Graph-based genome alignment and genotyping with HISAT2 and HISAT-genotype. Nature Biotechnology.

[46] Ashburner M, Ball CA, Blake JA, Botstein D, Butler H, Cherry JM (2000). Gene ontology: tool for the unification of biology. The Gene Ontology Consortium. Nature genetics.

[47] Liberzon A, Birger C, Thorvaldsdóttir H, Ghandi M, Mesirov JP, Tamayo P (2015). The Molecular Signatures Database (MSigDB) hallmark gene set collection. Cell Syst.

[48] Wu T, Hu E, Xu S, Chen M, Guo P, Dai Z (2021). clusterProfiler 4.0: A universal enrichment tool for interpreting omics data. Innovation (Cambridge (Mass)).

[49] Hänzelmann S, Castelo R, Guinney J (2013). GSVA: gene set variation analysis for microarray and RNA-seq data. BMC Bioinformatics.

[50] Chen Z, Quan L, Huang A, Zhao Q, Yuan Y, Yuan X (2018). seq-ImmuCC: Cell-Centric View of Tissue Transcriptome Measuring Cellular Compositions of Immune Microenvironment From Mouse RNA-Seq Data. Front Immunol.

[51] Therneau TM, Grambsch PM Modeling Survival Data: Extending the Cox Model. 2000.

[52] Gu Z (2022). Complex heatmap visualization. iMeta.

[53] Wang H, Guo M, Wei H, Chen Y (2023). Targeting p53 pathways: mechanisms, structures, and advances in therapy. Signal Transduction and Targeted Therapy.

[54] Owen KL, Brockwell NK, Parker BS (2019). JAK-STAT Signaling: A Double-Edged Sword of Immune Regulation and Cancer Progression. Cancers.

[55] Huynh J, Etemadi N, Hollande F, Ernst M, Buchert M (2017). The JAK/STAT3 axis: A comprehensive drug target for solid malignancies. Semin Cancer Biol.

[56] Yang X, Lin Y, Shi Y, Li B, Liu W, Yin W (2016). FAP Promotes Immunosuppression by Cancer-Associated Fibroblasts in the Tumor Microenvironment via STAT3-CCL2 Signaling. Cancer research.

[57] Yu H, Kortylewski M, Pardoll D (2007). Crosstalk between cancer and immune cells: role of STAT3 in the tumour microenvironment. Nat Rev Immunol.

[58] Zou S, Tong Q, Liu B, Huang W, Tian Y, Fu X (2020). Targeting STAT3 in Cancer Immunotherapy. Molecular cancer.

[59] Deek MP, Van der Eecken K, Sutera P, Deek RA, Fonteyne V, Mendes AA (2022). Long-Term Outcomes and Genetic Predictors of Response to Metastasis-Directed Therapy Versus Observation in Oligometastatic Prostate Cancer: Analysis of STOMP and ORIOLE Trials. Journal of clinical oncology: official journal of the American Society of Clinical Oncology.

[60] Hamid AA, Gray KP, Shaw G, MacConaill LE, Evan C, Bernard B (2019). Compound Genomic Alterations of TP53, PTEN, and RB1 Tumor Suppressors in Localized and Metastatic Prostate Cancer. European urology.

[61] Deek MP, Van der Eecken K, Phillips R, Parikh NR, Isaacsson Velho P, Lotan TL (2021). The Mutational Landscape of Metastatic Castration-sensitive Prostate Cancer: The Spectrum Theory Revisited. European urology.

[62] Donehower LA, Soussi T, Korkut A, Liu Y, Schultz A, Cardenas M (2019). Integrated Analysis of TP53 Gene and Pathway Alterations in The Cancer Genome Atlas. Cell reports.

[63] Kandoth C, McLellan MD, Vandin F, Ye K, Niu B, Lu C (2013). Mutational landscape and significance across 12 major cancer types. Nature.

[64] Rooney MS, Shukla SA, Wu CJ, Getz G, Hacohen N (2015). Molecular and genetic properties of tumors associated with local immune cytolytic activity. Cell.

[65] Tie Y, Tang F, Wei YQ, Wei XW (2022). Immunosuppressive cells in cancer: mechanisms and potential therapeutic targets. Journal of hematology & oncology.

[66] Lu C, Rong D, Zhang B, Zheng W, Wang X, Chen Z (2019). Current perspectives on the immunosuppressive tumor microenvironment in hepatocellular carcinoma: challenges and opportunities. Molecular cancer.

[67] Tie Y, Tang F, Wei Y-q, Wei X-w (2022). Immunosuppressive cells in cancer: mechanisms and potential therapeutic targets. Journal of hematology & oncology.

[68] Abdelnour SA, Xie L, Hassanin AA, Zuo E, Lu Y (2021). The Potential of CRISPR/Cas9 Gene Editing as a Treatment Strategy for Inherited Diseases. Frontiers in cell and developmental biology.

[69] Fu Y, He X, Gao XD, Li F, Ge S, Yang Z (2023). Prime editing: current advances and therapeutic opportunities in human diseases. Science bulletin.

[70] Kim MP, Li X, Deng J, Zhang Y, Dai B, Allton KL (2021). Oncogenic KRAS Recruits an Expansive Transcriptional Network through Mutant p53 to Drive Pancreatic Cancer Metastasis. Cancer Discov.

[71] Alvarado-Ortiz E, de la Cruz-López KG, Becerril-Rico J, Sarabia-Sánchez MA, Ortiz-Sánchez E, García-Carrancá A (2020). Mutant p53 Gain-of-Function: Role in Cancer Development, Progression, and Therapeutic Approaches. Frontiers in cell and developmental biology.

[72] Coradini D, Fornili M, Ambrogi F, Boracchi P, Biganzoli E (2012). TP53 mutation, epithelial-mesenchymal transition, and stemlike features in breast cancer subtypes. J Biomed Biotechnol.

[73] Dong P, Karaayvaz M, Jia N, Kaneuchi M, Hamada J, Watari H (2013). Mutant p53 gain-of-function induces epithelial-mesenchymal transition through modulation of the miR-130b-ZEB1 axis. Oncogene.

[74] Wen XM, Xu ZJ, Jin Y, Xia PH, Ma JC, Qian W (2021). Association Analyses of TP53 Mutation With Prognosis, Tumor Mutational Burden, and Immunological Features in Acute Myeloid Leukemia. Front Immunol.

[75] Pencik J, Philippe C, Schlederer M, Atas E, Pecoraro M, Grund-Gröschke S (2023). STAT3/LKB1 controls metastatic prostate cancer by regulating mTORC1/CREB pathway. Molecular cancer.

[76] Canesin G, Evans-Axelsson S, Hellsten R, Sterner O, Krzyzanowska A, Andersson T (2016). The STAT3 Inhibitor Galiellalactone Effectively Reduces Tumor Growth and Metastatic Spread in an Orthotopic Xenograft Mouse Model of Prostate Cancer. European urology.

[77] Luo J, Wang K, Yeh S, Sun Y, Liang L, Xiao Y (2019). LncRNA-p21 alters the antiandrogen enzalutamide-induced prostate cancer neuroendocrine differentiation via modulating the EZH2/STAT3 signaling. Nature communications.

[78] Johnson DE, O'Keefe RA, Grandis JR (2018). Targeting the IL-6/JAK/STAT3 signalling axis in cancer. Nat Rev Clin Oncol.

[79] Grivennikov SI, Greten FR, Karin M (2010). Immunity, inflammation, and cancer. Cell.

[80] Efe G, Dunbar KJ, Sugiura K, Cunningham K, Carcamo S, Karaiskos S (2023). p53 Gain-of-Function Mutation Induces Metastasis via BRD4-Dependent CSF-1 Expression. Cancer Discov.

[81] Phan TTT, Truong NV, Wu WG, Su YC, Hsu TS, Lin LY (2023). Tumor suppressor p53 mediates interleukin-6 expression to enable cancer cell evasion of genotoxic stress. Cell Death Discov.

[82] He G, Karin M (2011). NF-κB and STAT3 - key players in liver inflammation and cancer. Cell Res.

[83] Carnero A, Paramio JM (2014). The PTEN/PI3K/AKT Pathway in vivo, Cancer Mouse Models. Front Oncol.

[84] Cooks T, Pateras IS, Tarcic O, Solomon H, Schetter AJ, Wilder S (2013). Mutant p53 prolongs NF-κB activation and promotes chronic inflammation and inflammation-associated colorectal cancer. Cancer Cell.

[85] Xie J, Lin X, Deng X, Tang H, Zou Y, Chen W (2025). Cancer-associated fibroblast-derived extracellular vesicles: regulators and therapeutic targets in the tumor microenvironment. Cancer Drug Resist.

[86] Chen Y, Zhang M, Liu Z, Zhang N, Wang Q (2024). Ursodeoxycholic Acid Platinum(IV) Conjugates as Antiproliferative and Antimetastatic Agents: Remodel the Tumor Microenvironment through Suppressing JAK2/STAT3 Signaling. J Med Chem.

[87] Jung YY, Ha IJ, Um JY, Sethi G, Ahn KS (2022). Fangchinoline diminishes STAT3 activation by stimulating oxidative stress and targeting SHP-1 protein in multiple myeloma model. J Adv Res.

[88] Prestipino A, Emhardt AJ, Aumann K, O'Sullivan D, Gorantla SP, Duquesne S (2018). Oncogenic JAK2(V617F) causes PD-L1 expression, mediating immune escape in myeloproliferative neoplasms. Sci Transl Med.

[89] Li C, Li H, Zhang P, Yu LJ, Huang TM, Song X (2016). SHP2, SOCS3 and PIAS3 Expression Patterns in Medulloblastomas: Relevance to STAT3 Activation and Resveratrol-Suppressed STAT3 Signaling. Nutrients.

[90] Tonks NK (2005). Redox redux: revisiting PTPs and the control of cell signaling. Cell.

[91] Dustin CM, Heppner DE, Lin MJ, van der Vliet A (2020). Redox regulation of tyrosine kinase signalling: more than meets the eye. Journal of biochemistry.

[92] Wu C, Sun M, Liu L, Zhou GW (2003). The function of the protein tyrosine phosphatase SHP-1 in cancer. Gene.

[93] Tassidis H, Brokken LJ, Jirström K, Ehrnström R, Pontén F, Ulmert D (2010). Immunohistochemical detection of tyrosine phosphatase SHP-1 predicts outcome after radical prostatectomy for localized prostate cancer. Int J Cancer.

[94] Han Y, Amin HM, Franko B, Frantz C, Shi X, Lai R (2006). Loss of SHP1 enhances JAK3/STAT3 signaling and decreases proteosome degradation of JAK3 and NPM-ALK in ALK+ anaplastic large-cell lymphoma. Blood.

[95] Ahn KS, Sethi G, Sung B, Goel A, Ralhan R, Aggarwal BB (2008). Guggulsterone, a farnesoid X receptor antagonist, inhibits constitutive and inducible STAT3 activation through induction of a protein tyrosine phosphatase SHP-1. Cancer research.

[96] Tai WT, Cheng AL, Shiau CW, Liu CY, Ko CH, Lin MW (2012). Dovitinib induces apoptosis and overcomes sorafenib resistance in hepatocellular carcinoma through SHP-1-mediated inhibition of STAT3. Molecular cancer therapeutics.

[97] Sandur SK, Pandey MK, Sung B, Aggarwal BB (2010). 5-hydroxy-2-methyl-1,4-naphthoquinone, a vitamin K3 analogue, suppresses STAT3 activation pathway through induction of protein tyrosine phosphatase, SHP-1: potential role in chemosensitization. Molecular cancer research: MCR.

[98] Sadrkhanloo M, Paskeh MDA, Hashemi M, Raesi R, Motahhary M, Saghari S (2023). STAT3 signaling in prostate cancer progression and therapy resistance: An oncogenic pathway with diverse functions. Biomedicine & Pharmacotherapy.

