# Supplementary material for: Modulating the Immunosuppressive Tumor Microenvironment and Inhibiting Growth in Mutp53-Driven CRPC via STAT3 Pathway Blockade
Source: Int J Biol Sci. 2025 Apr 22;21(7):3081–98. doi: 10.7150/ijbs.111732 (PMC12080385; doi:10.7150/ijbs.111732)
Supplement: Supplementary file 1 — Supplementary figures and tables. [file ijbsv21p3081s1.zip › 111732n_supplementary_materials/Supplementary Figures/Supplementary Figure Legends.docx]

**Supplementary Figure Legends:**

**Supplementary Figure 1. Comparison of the average JAK/STAT3 pathway score across different mutation groups within the TCGA-PRAD cohort.** The groups analyzed included WTP53, p.R248Q, and other TP53 mutations, as well as a subgroup analysis of p.R248Q mutations in combination with other mutations.

**Supplementary Figure 2. Correlation analysis of SHP1 expression levels with immune-related factors in prostate cancer patients derived from the GSE25136 dataset (unpaired t test).**
