# Supplementary material for: Modulating the Immunosuppressive Tumor Microenvironment and Inhibiting Growth in Mutp53-Driven CRPC via STAT3 Pathway Blockade
Source: Int J Biol Sci. 2025 Apr 22;21(7):3081–98. doi: 10.7150/ijbs.111732 (PMC12080385; doi:10.7150/ijbs.111732)
Supplement: Supplementary file 1 — Supplementary figures and tables. [file ijbsv21p3081s1.zip › 111732n_supplementary_materials/Supplementary Tables/Supplementary Table 1.docx]

**Supplementary Table 1. TP53 hotspot mutations in the TCGA-PRAD cohort.**

| **Mutational site** | **Immune-Act** | **Immune-Sup** | **Non-Immune** | **Total mutation in Immune-Suppressed and Non-immune groups** |
| --- | --- | --- | --- | --- |
| **C124Wfs*25 (driver)** | 0 | 1 | 0 | 1 |
| **C135W (driver), HOMDEL (driver)** | 0 | 1 | 0 | 1 |
| **E285K (driver)** | 0 | 1 | 0 | 1 |
| **E298* (driver), Q144Gfs*24 (driver)** | 0 | 1 | 0 | 1 |
| **G266V (driver)** | 0 | 1 | 0 | 1 |
| **P177R (driver)** | 0 | 1 | 0 | 1 |
| **P82L, A74T** | 0 | 1 | 0 | 1 |
| **R249G (driver), HOMDEL (driver)** | 0 | 1 | 0 | 1 |
| **R282W (driver)** | 0 | 2 | 0 | 2 |
| **R337C (driver)** | 0 | 1 | 0 | 1 |
| **R342* (driver)** | 0 | 1 | 0 | 1 |
| **S149Pfs*21 (driver)** | 0 | 1 | 0 | 1 |
| **V173M (driver)** | 0 | 1 | 0 | 1 |
| **X33_splice (driver)** | 0 | 1 | 0 | 1 |
| **A86Vfs*55 (driver)** | 0 | 0 | 1 | 1 |
| **C135F (driver)** | 0 | 0 | 1 | 1 |
| **C135Y (driver)** | 0 | 0 | 1 | 1 |
| **C141G (driver), HOMDEL (driver)** | 0 | 1 | 1 | 2 |
| **C176R (driver)** | 0 | 0 | 1 | 1 |
| **E271V (driver), HOMDEL (driver)** | 0 | 0 | 1 | 1 |
| **G279E (driver), HOMDEL (driver)** | 0 | 0 | 1 | 1 |
| **H193R (driver)** | 0 | 0 | 1 | 1 |
| **M237I (driver)** | 0 | 0 | 1 | 1 |
| **N239D (driver)** | 0 | 1 | 1 | 2 |
| **Q165Hfs*17 (driver)** | 0 | 0 | 1 | 1 |
| **R248W (driver)** | 0 | 0 | 1 | 1 |
| **R273C (driver)** | 0 | 0 | 1 | 1 |
| **S90Ffs*53 (driver)** | 0 | 0 | 1 | 1 |
| **T256I (driver)** | 0 | 0 | 1 | 1 |
| **V157F (driver), G199V (driver)** | 0 | 0 | 1 | 1 |
| **V203Wfs*44 (driver)** | 0 | 0 | 1 | 1 |
| **X126_splice (driver)** | 0 | 2 | 1 | 3 |
| **X307_splice (driver)** | 0 | 0 | 1 | 1 |
| **X331_splice (driver)** | 0 | 0 | 1 | 1 |
| **Y163H (driver)** | 0 | 0 | 1 | 1 |
| **Y163H (driver), HOMDEL (driver)** | 0 | 0 | 1 | 1 |
| **G245D (driver)** | 0 | 1 | 2 | 3 |
| **R209Kfs*6 (driver)** | 0 | 0 | 2 | 2 |
| **R248Q (driver)** | 0 | 1 | 3 | 4 |
| **R175H (driver)** | 1 | 1 | 0 | 2 |
