# Supplementary material for: Modulating the Immunosuppressive Tumor Microenvironment and Inhibiting Growth in Mutp53-Driven CRPC via STAT3 Pathway Blockade
Source: Int J Biol Sci. 2025 Apr 22;21(7):3081–98. doi: 10.7150/ijbs.111732 (PMC12080385; doi:10.7150/ijbs.111732)
Supplement: Supplementary file 1 — Supplementary figures and tables. [file ijbsv21p3081s1.zip › 111732n_supplementary_materials/Supplementary Tables/Supplementary Table 2.docx]

**Supplementary Table 2. Plasmids and primers utilized in the present study.**

| **Primer_ID** | **Sequence** |
| --- | --- |
| RM-1 CRISPR/cas9 (Oligo) | AGTATACCACCATCCACTACAAGTACATGTGTAATAGCTCCTGTATGGGGGGCATGAACCAGCGACCTATCCTTACCATCATCACACTGGAAGACTCCAGgtaggaaggcgcgtggtaggt |
| RM-1 CRISPR/cas9 gRNA-1 | GCGGTTCATGCCCCCCATGCAGG |
| RM-1 CRISPR/cas9 gRNA-2 | TGTAATAGCTCCTGCATGGGGGG |
| C4-2 KO *TP53* Oligo | GACTGTACCACCATCCACTACAACTACATGTGTAACAGTTCCTGCATGGGCGGTATGAACCAGAGGCCCATCCTCACCATCATCACACTGGAAGACTCCAGgtcaggagccacttgccacc |
| C4-2 KO *TP53* gRNA-1 (Forward) | GCAGTCACAGCACATGACGGAGG |
| C4-2 KO *TP53* gRNA-2 (Forward) | GAGCGCTGCTCAGATAGCGATGG |
| C4-2 KO *TP53* gRNA-3 (Reverse) | ATCTGAGCAGCGCTCATGGTGGG |
| Si*SHP1*-1 | Forward: GATCCAAAAAAGGGATCAGGTGACCCATATTCTCTCTTGAAGAATATGGGTCACCTGATCCC;  Reverse: CACCGGGATCAGGTGACCCATATTCTTCAAGAGAGAATATGGGTCACCTGATCCCTTTTTTG |
| Si*SHP1*-2 | Forward: GATCCAAAAAAGCATCGCCCAGTTCATTGAAACTCTCTTGAAGTTTCAATGAACTGGGCGATGC;  Reverse: CACCGCATCGCCCAGTTCATTGAAACTTCAAGAGAGTTTCAATGAACTGGGCGATGCTTTTTTG |
| Si*SHP1*-3 | Forward: GATCCAAAAAAGATTCGGGAGATCTGGCATTATCTCTTGAATAATGCCAGATCTCCCGAATC;  Reverse: CACCGATTCGGGAGATCTGGCATTATTCAAGAGATAATGCCAGATCTCCCGAATCTTTTTTG |
| Si*TP53*-1 | Forward: CACCAUCCACUACAACUACAU  Reverse: AUGUAGUUGUAGUGGAUGGUG |
| Si*TP53*-2 | Forward: UCAGACCUAUGGAAACUACUU  Reverse: AAGUAGUUUCCAUAGGUCUGA |
