# Supplementary material for: Modulating the Immunosuppressive Tumor Microenvironment and Inhibiting Growth in Mutp53-Driven CRPC via STAT3 Pathway Blockade
Source: Int J Biol Sci. 2025 Apr 22;21(7):3081–98. doi: 10.7150/ijbs.111732 (PMC12080385; doi:10.7150/ijbs.111732)
Supplement: Supplementary file 1 — Supplementary figures and tables. [file ijbsv21p3081s1.zip › 111732n_supplementary_materials/Supplementary Tables/Supplementary Table 3.docx]

**Supplementary Table 3. Antibodies utilized in the current study.**

| **Antibodies** | **Sources** |
| --- | --- |
| Phospho-Jak2 (Tyr1007/1008) Rabbit mAb | Cell Signaling Technology (#3776) |
| Jak2 Rabbit mAb Antibody | Cell Signaling Technology (#3230) |
| Stat3 Rabbit mAb Antibody | Cell Signaling Technology (# 12640) |
| Stat3 Mouse mAb Antibody | Cell Signaling Technology (# 9139) |
| Phospho-Stat3 (Tyr705) Rabbit mAb Antibody | Cell Signaling Technology (# 9145) |
| Phospho-Stat3 (Tyr705) Mouse mAb Antibody | Cell Signaling Technology (# 4113) |
| Rabbit polyclonal antibody to p53 | Affinity (#AF0879) |
| Mouse monoclonal antibody to p53 | Affinity (#BF8013) |
| HA-Tag Rabbit mAb | Cell Signaling Technology (# 3724) |
| YKDDDDK Tag (D6W5B) Rabbit mAb Antibody | Cell Signaling Technology (# 14793) |
| HA Tag Monoclonal antibody (mouse) | Proteintech (#66006-2-Ig) |
| DYKDDDDK tag Monoclonal antibody (mouse) | Proteintech (#66008-4-Ig) |
| Rabbit polyclonal antibody to GAPDH | Affinity (#AF7021) |
| Anti-mouse CD3 | BD Bioscience (# 553066) |
| Anti-mouse CD8 | BD Bioscience (# 551162) |
| Anti-mouse F4/80 | Elabscience (# E-AB-F0995E) |
| Anti-mouse CD11b | Biolegend (#101206) |
| Anti-mouse CD86 | BD Bioscience (#565250) |
| Anti-mouse CD206 | BD Bioscience (#568807) |
| Anti-mouse IFN-γ | BD Bioscience (#554412) |
