# Supplementary material for: Modulating the Immunosuppressive Tumor Microenvironment and Inhibiting Growth in Mutp53-Driven CRPC via STAT3 Pathway Blockade
Source: Int J Biol Sci. 2025 Apr 22;21(7):3081–98. doi: 10.7150/ijbs.111732 (PMC12080385; doi:10.7150/ijbs.111732)
Supplement: Supplementary file 1 — Supplementary figures and tables. [file ijbsv21p3081s1.zip › 111732n_supplementary_materials/Supplementary Tables/Supplementary Table 4.docx]

**Supplementary Table 4. Details of the 19 studies included in the current research.**

| **Author(s) and year of publication** | **Data Source** | **PMID** |
| --- | --- | --- |
| Barry S Taylor *et al.* 2010 | Cancer Cell | 20579941 |
| Kristin L Granlund *et al.* 2020 | Cell metabolism | 31564440 |
| Shancheng Ren *et al.* 2017 | European urology | 28927585 |
| Christopher E Barbieri *et al.* 2012 | Nature genetics | 22610119 |
| Clarissa Gerhauser *et al.* 2018 | Cancer Cell | 30537516 |
| Sylvan C Baca *et al.* 2013 | Cell | 23622249 |
| MD Anderdson Cancer Center. 2016 | <https://www.cbioportal.org/study/clinicalData?id=prad_tcga> | / |
| Joshua Armenia *et al.* 2018 | Nature genetics | 29610475 |
| Himisha Beltran *et al.* 2016 | Nature medicine | 26855148 |
| Bastien Nguyen *et al.* 2020 | European urology | 32317181 |
| Konrad H Stopsack *et al.* 2021 | Clinical cancer research | 34667026 |
| Konrad H Stopsack *et al.* 2020 | Clinical cancer research | 32220891 |
| Wassim Abida *et al.* 2017 | JCO precision oncology | 28825054 |
| Akash Kumar *et al.* 2016 | Nature medicine | 26928463 |
| Wassim Abida *et al.* 2019 | Proc Natl Acad Sci U S A | 31061129 |
| MPCproject Data，2021 | <https://www.cbioportal.org/study/summary?id=mpcproject_broad_2021> | / |
| Dan Robinson *et al.* 2015 | Cell | 26000489 |
| Catherine S Grasso *et al.* 2012 | Nature | 22722839 |
| Dong Gao *et al.* 2014 | Cell | 25201530 |
