# Supplementary material for: Modulating the Immunosuppressive Tumor Microenvironment and Inhibiting Growth in Mutp53-Driven CRPC via STAT3 Pathway Blockade
Source: Int J Biol Sci. 2025 Apr 22;21(7):3081–98. doi: 10.7150/ijbs.111732 (PMC12080385; doi:10.7150/ijbs.111732)
Supplement: Supplementary file 1 — Supplementary figures and tables. [file ijbsv21p3081s1.zip › 111732n_supplementary_materials/Supplementary Tables/Supplementary Table 5.docx]

**Supplementary Table 5. Summary of studies included in the meta-analysis.**

| **Observed Outcome** | **TP53 type** | **Tumor type** | **Source** | **Link** | **Sample size** |
| --- | --- | --- | --- | --- | --- |
| PFS | TP53 mutation | NA | blood sample | <https://pubmed.ncbi.nlm.nih.gov/34743450/> | 239 samples |
| PFS | TP53 mutation | NA | blood sample |  |  |
| PFS | TP53 mutation | NA | blood sample |  |  |
| event-free survival (EFS) defined as time to prostate-specific antigen relapse, metastasis, or death; | TP53 genomic alteration | LCSPC | formalin-fixed, paraffin-embedded (FFPE) tissue from biopsies of patients with localized or metastatic disease | <https://pubmed.ncbi.nlm.nih.gov/30553611/> | 285 samples |
| MFS | TP53 mutation | NA | formalin-fixed paraffin-embedded (FFPE) block | <https://pubmed.ncbi.nlm.nih.gov/32896505/> | 493 samples |
| bPFS | TP53 genomic alteration | mCRPC | blood sample | https://pubmed.ncbi.nlm.nih.gov/30209161/ | 168 samples |
| bPFS | TP53 mutation | mHSPC | Tumor biopsy specimens | <https://pubmed.ncbi.nlm.nih.gov/33727260/> | 60 samples |
| PFS (included radiographic PFS, clinical progression or death) | TP53 alteration | mCRPC | blood sample | <https://pubmed.ncbi.nlm.nih.gov/33794293/> | 151 samples |
| rPFS | TP53 mutation | mCSPC | 269 samples were from primary tumor and 25 samples were from metastatic sites | <https://pubmed.ncbi.nlm.nih.gov/33419682/> | 269 samples were from primary tumor and 25 samples were from metastatic sites |
