# Supplementary material for: Modulating the Immunosuppressive Tumor Microenvironment and Inhibiting Growth in Mutp53-Driven CRPC via STAT3 Pathway Blockade
Source: Int J Biol Sci. 2025 Apr 22;21(7):3081–98. doi: 10.7150/ijbs.111732 (PMC12080385; doi:10.7150/ijbs.111732)
Supplement: Supplementary file 1 — Supplementary figures and tables. [file ijbsv21p3081s1.zip › 111732n_supplementary_materials/Supplementary Tables/Supplementary Table 6.docx]

Supplementary Table 6. Differentially Expressed Genes Between Mutant p53 (Mutp53) and Wild-type p53 (WTp53) Tumor Groups.

| Gene ID | Fold change | log_2_(Fold change) | p-Value |
| --- | --- | --- | --- |
| *Ppp1r9a* | 0.032487723 | -4.943961542 | 1.71E-12 |
| *Gabre* | 0.049464107 | -4.337474161 | 2.81E-11 |
| *Slitrk5* | 0.036376302 | -4.780857322 | 3.68E-11 |
| *Dlgap1* | 0.010326982 | -6.597437541 | 4.78E-11 |
| *Casd1* | 0.110385492 | -3.17937753 | 1.40E-10 |
| *Trp53* | 0.323867006 | -1.626526594 | 2.25E-10 |
| *Tert* | 0.019293408 | -5.695748196 | 3.33E-10 |
| *Lingo2* | 0.102190762 | -3.290663308 | 3.44E-10 |
| *Arhgef15* | 4.205771673 | 2.07237053 | 3.66E-10 |
| *Tmlhe* | 0.09529524 | -3.391452035 | 3.84E-10 |
| *Sdr39u1* | 0.130890751 | -2.933564935 | 5.99E-10 |
| *Abhd6* | 0.26448197 | -1.91875872 | 1.10E-09 |
| *Fgf10* | 0.040430066 | -4.628427617 | 1.65E-09 |
| *Pde3b* | 0.203683729 | -2.29559736 | 2.85E-09 |
| *Eda2r* | 0.162978523 | -2.617246237 | 6.50E-09 |
| *Prkar1b* | 0.11714957 | -3.093576433 | 6.90E-09 |
| *Fat4* | 0.158452009 | -2.657882145 | 8.22E-09 |
| *Cntln* | 0.088882583 | -3.491955445 | 8.50E-09 |
| *Tmem74* | 0.266752346 | -1.906427136 | 9.39E-09 |
| *Patj* | 0.223014979 | -2.164787479 | 1.06E-08 |
| *Trp53i11* | 4.156685501 | 2.055433596 | 1.53E-08 |
| *Cdkn1c* | 0.198784625 | -2.330721917 | 3.06E-08 |
| *Kif26a* | 5.436651271 | 2.442718291 | 3.28E-08 |
| *Grb10* | 0.258633792 | -1.95101731 | 4.06E-08 |
| *Mpp3* | 3.552675421 | 1.828905887 | 5.56E-08 |
| *Dnajc22* | 8.885873379 | 3.151513583 | 5.58E-08 |
| *Gm42346* | 0.028747423 | -5.120423566 | 5.67E-08 |
| *Gja3* | 5.837545556 | 2.545361904 | 6.01E-08 |
| *Tubb2a* | 0.255945557 | -1.96609113 | 6.13E-08 |
| *Cth* | 0.063041307 | -3.987558738 | 7.02E-08 |
| *Arsj* | 0.073358624 | -3.768889604 | 7.29E-08 |
| *Nol3* | 0.125782379 | -2.990998266 | 7.36E-08 |
| *Bank1* | 0.28017769 | -1.835586012 | 1.00E-07 |
| *Cped1* | 0.214882903 | -2.218377395 | 1.24E-07 |
| *Larp6* | 0.151069607 | -2.72671465 | 1.37E-07 |
| *Mmp2* | 0.229804088 | -2.12152363 | 1.41E-07 |
| *Foxred2* | 0.204165437 | -2.292189441 | 1.50E-07 |
| *Rps6ka6* | 0.026078837 | -5.260976658 | 1.56E-07 |
| *Ina* | 0.083371294 | -3.584305467 | 1.63E-07 |
| *Nnmt* | 0.223520391 | -2.161521645 | 2.08E-07 |
| *Mgp* | 0.20829457 | -2.263302862 | 2.19E-07 |
| *Zfp385b* | 0.178756633 | -2.483931322 | 2.20E-07 |
| *Thsd4* | 2.969945193 | 1.570436308 | 2.22E-07 |
| *Oplah* | 0.264591591 | -1.918160881 | 2.48E-07 |
| *Csmd3* | 0.1696777 | -2.559131123 | 2.78E-07 |
| *Gpr149* | 0.101105481 | -3.306066881 | 2.92E-07 |
| *Acacb* | 3.112342506 | 1.638000834 | 3.17E-07 |
| *Scn8a* | 16.47137561 | 4.041889142 | 3.20E-07 |
| *Scube3* | 0.071620699 | -3.803479599 | 3.48E-07 |
| *Samd5* | 0.242002991 | -2.046903216 | 3.50E-07 |
