# Supplementary figures and images for: Modulating the Immunosuppressive Tumor Microenvironment and Inhibiting Growth in Mutp53-Driven CRPC via STAT3 Pathway Blockade
Source: Int J Biol Sci. 2025 Apr 22;21(7):3081–98. doi: 10.7150/ijbs.111732 (PMC12080385; doi:10.7150/ijbs.111732)

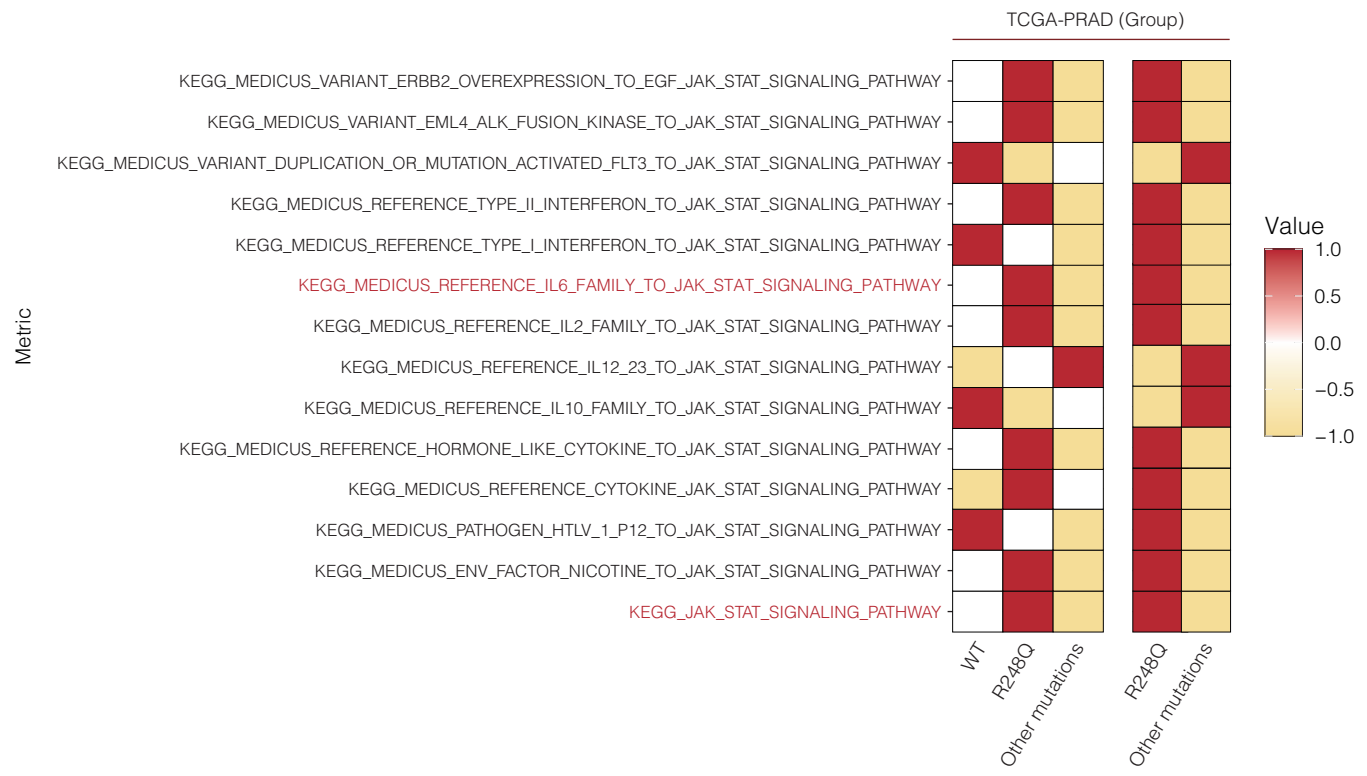

Supplement: Supplementary file 1 — Supplementary figures and tables. [file ijbsv21p3081s1.zip › 111732n_supplementary_materials/Supplementary Figures/Supplematary Figure 1.pdf]

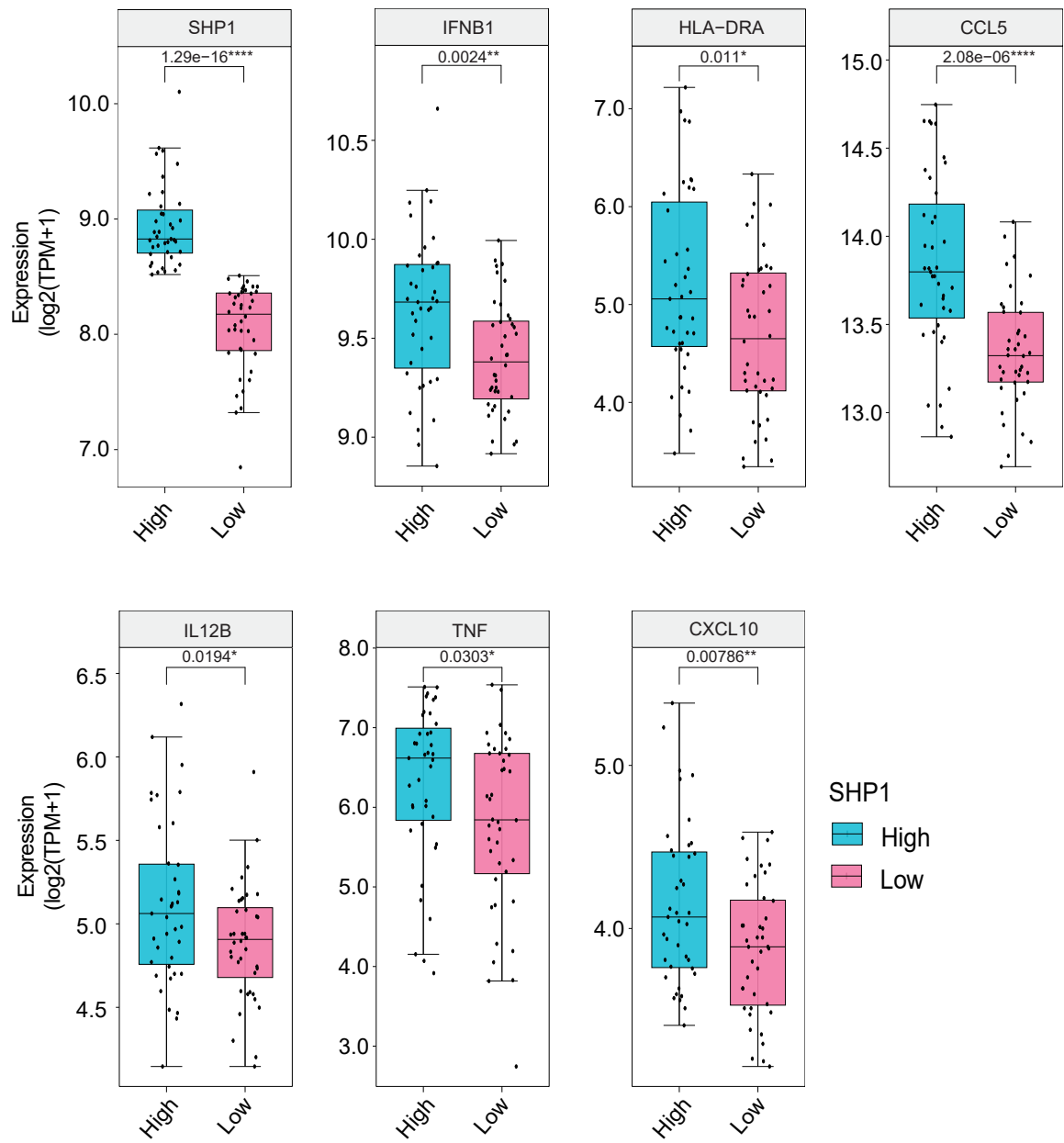

Supplement: Supplementary file 1 — Supplementary figures and tables. [file ijbsv21p3081s1.zip › 111732n_supplementary_materials/Supplementary Figures/Supplementary Figure 2.pdf]
